# Responding to the ECHO trial results: modelling the potential impact of changing contraceptive method mix on HIV and reproductive health in South Africa

**DOI:** 10.1002/jia2.25620

**Published:** 2020-10-08

**Authors:** Jennifer A Smith, Leo Beacroft, Fareed Abdullah, Buyile Buthelezi, Manala Makua, Chelsea Morroni, Gita Ramjee, Claudia Velasquez, Timothy B. Hallett

**Affiliations:** ^1^ MRC Centre for Global Infectious Disease Analysis Department of Infectious Disease Epidemiology Imperial College London London United Kingdom; ^2^ Office of AIDS and TB Research South African Medical Research Council Pretoria South Africa; ^3^ USAID Pretoria South Africa; ^4^ National Department of Health Pretoria South Africa; ^5^ Botswana Harvard AIDS Institute Gaborone Botswana; ^6^ Liverpool School of Tropical Medicine Liverpool United Kingdom; ^7^ University of Cape Town Cape Town South Africa; ^8^ Aurum Institute Parktown South Africa; ^9^ UNAIDS Eastern and Southern Africa Johannesburg South Africa

**Keywords:** HIV, hormonal contraception, theoretical models, pregnancy complications, women, South Africa

## Abstract

**Introduction:**

Some observational data suggest that the progestogen injectable contraceptive depot medroxyprogesterone acetate (DMPA) may increase a woman’s risk of HIV acquisition but a randomized clinical trial did not find a statistically significant increase in HIV risk for women using DMPA compared to two other methods. However, it could not rule out up to 30% increased HIV risk for DMPA users. We evaluate changes to contraceptive method mix in South Africa under different assumptions about the existence and strength of a possible undetected relationship between DMPA use and HIV risk.

**Methods:**

A mathematical model was developed to simulate the ongoing HIV epidemic and contraceptive method mix in South Africa to estimate how changes in method mix could impact HIV‐ and reproductive health‐related outcomes. We made different assumptions about the relationship between DMPA use and HIV risk, from no relationship to a 30% increase in HIV risk for women using DMPA. Scenario analyses were used to investigate the impact of switching away from DMPA predominance to new patterns of contraceptive use.

**Results:**

In South Africa, the HIV‐related benefits of reduced DMPA use could be as great as the harms of increased adverse reproductive health outcomes over 20 years, if DMPA did increase the risk of HIV acquisition by a relative hazard of infection of 1.1 or greater. A reduction in DMPA use among HIV‐positive women would have no benefit in terms of HIV infections, but would incur additional negative reproductive health outcomes. The most important driver of adverse reproductive health outcomes is the proportion of women who switch away from DMPA to no contraceptive method.

**Conclusions:**

If there is any real increased HIV risk for DMPA users that has not been detected by the recent randomized trial, a reduction in DMPA use could reduce the ongoing number of new HIV infections. However, such a change would place more women at risk of adverse reproductive health effects. It is imperative that these effects are minimized by focusing on expanding access to safe, effective and acceptable alternative contraceptive methods for all women.

## 1 INTRODUCTION

Increasing access to modern contraceptive methods has been vital to global development over the past 30 years [1, 2]. The use of safe and effective methods of contraception gives women control over the number and timing of their pregnancies, and so reduces maternal morbidity and mortality and increases newborn and child survival rates [3, 4]. However, concerns have been raised over the use of the popular progestogen‐based intramuscular injectable depot medroxyprogesterone acetate (DMPA) due to evidence from some observational studies suggesting that it increases a woman’s risk of HIV acquisition. A meta‐analysis of the highest quality observational studies reported a 40% increase in HIV acquisition rates for women using DMPA compared to women not using hormonal methods (hazard ratio (HR) = 1.40, 95% confidence interval [CI] = 1.24 to 1.48) [5].

Recently, the “Evidence for Contraceptive Options and HIV Outcomes” (ECHO) randomized open‐label trial did not find a statistically significant increase in HIV acquisition risk for women using intramuscular DMPA compared to those using the copper intrauterine device (IUD) or the levonorgestrol (LNG) implant [6]. In response to the new evidence, the World Health Organization (WHO) changed the medical eligibility criteria (MEC) classification for progestogen‐based injectables (including both DMPA and a second progestogen‐based injectable, norethisterone enanthate [NET‐EN]), from a “2,” signifying that the "benefits generally outweigh theoretical or proven risks" to a “1” (“no restriction for the use of the contraceptive method”), noting that “new high‐quality evidence supersedes the low to low‐moderate quality evidence from observational studies that had been previously available to inform WHO’s guidance” [7].

However, some have argued that a lack of statistical significance for a positive interaction in the ECHO trial does not preclude the possibility of some effect of DMPA on HIV acquisition, merely that it reduces the chance of a strong association [8, 9, 10, 11], because the trial was not designed to detect a hazard ratio below 1.5 (and post‐trial analyses revealed that an observed HR of 1.3 or above would have been statistically significant, due to higher than expected subject retention and HIV incidence) [12, 13]. Furthermore, for ethical reasons, the trial provided a comparison of HIV risk among DMPA users versus users of other existing contraceptives, rather than no contraceptive.

A Bayesian interpretation would synthesize the new evidence with the existing evidence, weighting different types of evidence according to the rigour of their study design. This approach could, unfortunately, result in the possibility of a small but real effect of DMPA use on HIV acquisition, which would substantially affect a woman’s absolute HIV risk in areas of high HIV incidence and high DMPA use.

If DMPA does increase HIV risk to some extent, continued use could lead to additional HIV infections. However, a reduction in DMPA use to avert possible excess HIV infections could lead to additional unintended pregnancies due to non‐use of contraception or switching to a less effective method, with the potential for an increase in negative downstream repercussions – for example morbidity and mortality related to pregnancy. The benefits and risks of either option depend on the interplay of HIV epidemiology, patterns of contraceptive use, quality of pregnancy and abortion care, and the magnitude of any real effect of DMPA on HIV risk.

South Africa is among the countries with both the highest DMPA use and HIV incidence [14, 15]. Injectables are the most commonly used contraceptive, representing 47% of the modern contraceptive method mix. In 2016, 23% of all women used an injectable method, of whom 16% used DMPA and 7% used NET‐EN [16]. DMPA use was highest among 25 to 34 year‐olds. HIV incidence in South Africa was 0.93 (0.71 to 1.11) and 1.51 (1.31 to 1.71) per 100 person‐years among 15‐ to 49‐ and 15‐ to 24‐year‐old women, respectively, in 2017, and 3.81 (3.45 to 4.21) per 100 person‐years among the women enrolled in ECHO [6, 17].

The United States Agency for International Development (USAID) and Imperial College London convened a meeting with leading South African academics, policy makers and other key stakeholders in January 2018 to develop a set of mathematical modelling analyses in advance of the ECHO trial results that would help inform any policy change needed in the event that ECHO provided strong evidence for an association between DMPA use and HIV risk. Although a policy change is unlikely in light of the trial results and subsequent revision to WHO guidelines, careful consideration should be paid to the possibility that the trial did not detect a smaller association between DMPA use and HIV risk. Here we present the results of those analyses for a range of HRs that are consistent with both the ECHO trial results and prior observational evidence.

## 2 METHODS

We calibrated a deterministic dynamic transmission model to represent the ongoing HIV epidemic and changing contraceptive method mix over time in South Africa using data on age‐ and sex‐specific HIV prevalence, total HIV incidence, incidence among high‐risk women, and age‐specific contraceptive method mix [18, 19]. The model has been described in detail elsewhere and full details are provided in the Supporting Information [20, 21]. Key features of the model include representation of the population age structure, sexual behaviour (sex acts, condom use, partner change rates), HIV transmission and natural history, rollout of the antiretroviral therapy (ART) cascade, prevention interventions (male circumcision, expanded condom use), contraceptive method mix and reproductive health outcomes.

We assigned women to a type of contraception based on current method mix by age, and assumed that this pattern of use has held constant since the beginning of the HIV epidemic (Table 1). Many methods, including injectables, oral pills and female sterilization, have been used at broadly similar levels since the first Demographic and Health Survey in 1998, for example injectables (both DMPA and NET‐EN) were used by 27% of all women in 1998 and 23% in 2016 [16, 22]. We parameterized the effectiveness of each method assuming the quoted “typical use” contraceptive efficacies and one‐year continuation rates, recognizing the limitation that in reality “typical use” could be country specific [18, 23].

**Table 1:** Model contraceptive parameters

Contraceptive	Modelled prevalence among 15 to 49 year‐old women in 2012	Efficacy (typical use) [23]	Assumed increase in HIV risk
No method	51.4%	15%	1
Combined oral contraceptive	8.6%	91%	1
DMPA	14.5%	94%	Varied from 1 to 1.3
NET‐EN	10.4%	94%^a^	1
Copper IUD	1.7%	99.2%	1
Female sterilization	6.6%	99.5%	1
Implant	0%	99.5%	1
Other methods	6.7%	83.9%^b^	1

^a^ Assumed to be the same as DMPA

^b^ average of the efficacies of male sterilization, withdrawal, fertility awareness and male condoms.

Model outputs include demographic, HIV‐related and reproductive health outcomes encompassing morbidities and mortality stemming from both HIV infection and unintended pregnancy. All modelled health outcomes are jointly summarized as disability‐adjusted life‐years (DALYs). For HIV‐related outcomes, these include the different stages of HIV infection and ART use, and for reproductive health outcomes, these include morbidities associated with unsafe abortion and complications of labour (haemorrhage, puerperal sepsis, eclampsia, obstructed labour), and mortality resulting from unsafe abortion and maternal deaths. All model simulations were run for 20 years and the results summed for that period without discounting.

Two sources of uncertainty are included in the model outputs. First, several parameters defining the HIV epidemic were fitted (per sex act probability of HIV transmission, sexual mixing rates between different behavioural risk groups, the size of these risk groups and the start time of the epidemic) by running the model 20,000 times and using a filtration method to select the 100 most acceptable epidemic fits, and the analysis was repeated sampling from each of these parameter sets. Second, we sample the maternal mortality ratio (MMR) in South Africa from a log‐normal distribution constructed using a point estimate and associated 95% CI [24].

The association between DMPA use and HIV acquisition risk in the model was varied between an HR of 1.0 (representing no association, consistent with the ECHO trial results) to 1.3 (representing a 30% increase in HIV risk for women using DMPA, consistent with both the ECHO trial results and a meta‐analysis of observational data [5, 6]) at increments of 0.1, and all analyses were repeated under each assumption. We do not consider the possibility that HR < 1 because this would not result in any undetected ongoing excess HIV risk. In the absence of data, we assume that the IUD and levonorgestrel implant have no effect on risk of acquiring HIV. Similarly, we assume that NET‐EN does not affect HIV acquisition risk.

The analysis compares the HIV‐ and reproductive health‐related outcomes in a 20‐year period that could result from changes in the contraceptive method mix from 2019. Eighteen different scenarios, described in Table 2, were defined by stakeholders that vary in respect of:

- the magnitude of migration away from DMPA (a “soft” change is defined as a decrease in new DMPA users, but all current DMPA users continuing).
- whether migration is limited to HIV‐negative women or also includes HIV‐positive women (a “medium” change is defined as when all HIV‐negative women stop using DMPA, but HIV‐positive women can continue to use it; a “hard” change is defined as when all women, HIV positive and negative, stop using DMPA).
- the choice of replacement contraceptive – this can be an alternative method of comparable effectiveness (and no associated HIV risk), implant, no method, or some combination of these.

**Table 2:** Analysis plan

A) Magnitude of migration away from DMPA use:				
	Potential new starters (HIV negative)	Potential new starters (HIV positive)	Already using DMPA (HIV negative)	Already using DMPA (HIV positive)
1. “Soft change”	50% reduction in DMPA uptake	No change in DMPA uptake	Continue using DMPA	
2. “Medium change”	Do not start DMPA	No change in DMPA uptake	Stop using DMPA	Continue using DMPA
3. “Hard change”	Do not start DMPA		Stop using DMPA	

B) Contraceptive replacement for those stopping using DMPA:
1. 100% switch to an alternative method of comparable efficacy and no associated HIV risk
2. 100% switch to implant
3. 90% redistribute among other methods proportionately; 10% move to no method
4. 80% redistribute among other methods proportionately; 20% move to no method
5. 90% redistribute among other methods disproportionately in favour of NET‐EN (OR = 2); 10% move to no method
6. 90% redistribute among other methods disproportionately in disfavour of NET‐EN (OR = 0.5); 10% move to no method

Eighteen different scenarios are constructed by combining each of the three magnitudes of migration options (Panel A) with the six contraceptive replacement options (Panel B). DMPA is replaced over three years from 2019 onwards. Baseline: no change in DMPA use.

These scenarios are not intended to predict what will happen, but rather to illustrate key relationships and their potential outcomes over a wide range of assumptions. For example it is unlikely that DMPA availability would ever be restricted for HIV‐positive women, who have limited alternative contraceptive options [25].

Finally, we used an analysis of the 2016 South African HIV Investment Case to infer the cost at which each scenario would be cost‐effective [26]. That analysis implied that HIV interventions that lead to saving life‐years at a cost of $547 to 872 per life‐year saved (LYS) would be at the margin of cost‐effectiveness based on the set of interventions being funded under the current budget. We therefore multiplied the net health impact (in LYS) of each scenario by the cost range above to give the total affordable cost of an intervention to implement each switching scenario.

## 3 RESULTS

**Figure 1:**
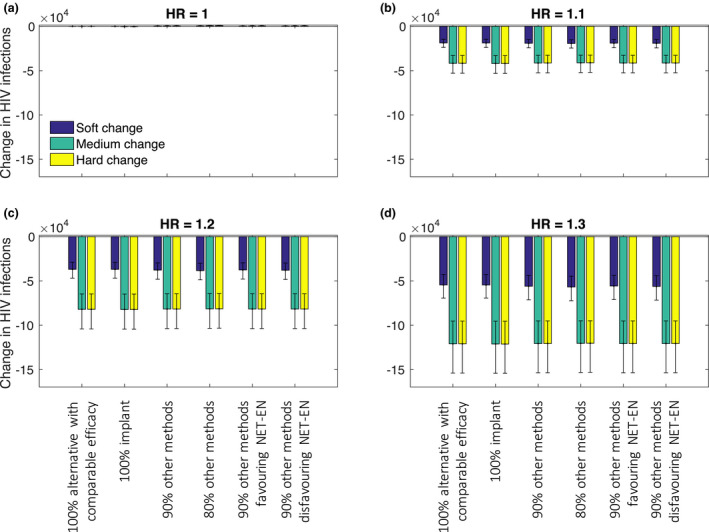
Change in HIV infections among women over 20 years under different assumed HRs for DMPA‐HIV risk association and different switching assumptions. Uncertainty intervals represent 90% of variability in model outputs.

**Figure 2:**
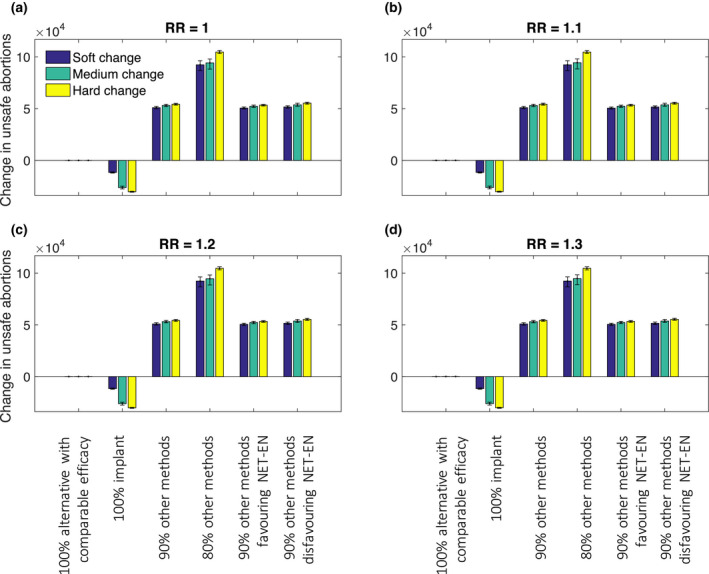
Change in unsafe abortions over 20 years under different assumed HRs for DMPA‐HIV risk association and different switching assumptions. Uncertainty intervals represent 90% of variability in model outputs.

**Figure 3:**
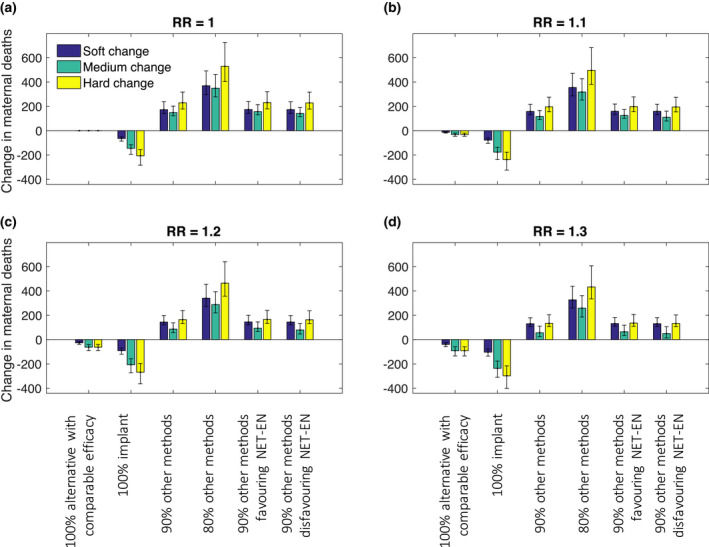
Change in maternal deaths over 20 years under different assumed HRs for DMPA‐HIV risk association and different switching assumptions. Uncertainty intervals represent 90% of variability in model outputs.

Figures 1, 2, 3 show the changes in the numbers of HIV infections among women, unsafe abortions and maternal deaths over a 20‐year period, following each of the changes in the contraceptive method mix (Table 2), under a range of different assumptions for a possible association between HIV risk and DMPA use.

### 3.1 HIV infections

If there is no association between DMPA use and HIV risk, the proportion of women ceasing to use DMPA has no impact on the expected number of HIV infections (Figure 1A). However, when the HR is assumed to be greater than one, reduced DMPA use leads to a reduction in HIV infections (Figure 1B‐D). The magnitude of the reduction depends on: (i) the strength of the risk association assumed – the greater the HR, the greater the reduction in HIV infections when women cease to use DMPA; and, (ii) the number of HIV‐negative women who discontinue DMPA use or do not initiate DMPA use (the “soft” versus the “medium” and “hard” reductions). For example for a HR of 1.1, 14,600 to 53,200 fewer HIV infections would occur over 20 years among women (0.7% to 2.2% of projected total infections among women in this period), depending on the number of women switching away from DMPA. The assumption regarding the replacement contraceptive(s) does not affect the HIV infections averted, and there is no additional impact from reducing DMPA use among HIV‐positive women (medium vs. hard change scenario). In all cases, the number of HIV infections averted increases over time (Figures S13‐S14).

### 3.2 Unsafe abortions

In contrast to HIV infections, the strength of an association between DMPA use and HIV risk has no impact on the number of unsafe abortions following a change in DMPA use. Instead, this depends on the mix of replacement contraceptive(s) and particularly the proportion of women (irrespective of HIV status) who stop using any method (Figure 2). For example if all DMPA users were to switch to an alternative method with comparable effectiveness and no associated HIV risk (contraceptive replacement option 1), there would be no change in unsafe abortions because both methods have the same effectiveness. If all ex‐DMPA users switch to the implant (replacement 2), 10,700 to 30,700 (2.0% to 5.5%) fewer unsafe abortions would occur over 20 years because the implant is a more effective contraceptive. But if some proportion of DMPA users switch to no method, we anticipate an increase in unsafe abortions over 20 years regardless of the mix of other methods in use – 49,300 to 56,400 (9.1% to 10.2%) extra with 10% DMPA users moving to no method (replacement 3, 5 and 6) and 86,700 to 106,000 (15.9% to 19.2%) extra with 20% moving to no method (replacement 4). The change in unsafe abortions is greater with more women moving away from DMPA and also increases over time (Figures S15‐S16).

### 3.3 Maternal deaths

If DMPA use was reduced, the impact on maternal deaths would depend on both the true association between DMPA use and HIV acquisition risk and the contraceptive replacement option(s). If there is no effect (HR = 1), the pattern of change in maternal deaths is similar to that for unsafe abortions, with the exception that a medium change is the least harmful when switching to a mix of existing methods and no method (Figure 3A). The differences between medium and soft/hard change become more pronounced as the magnitude of the assumed HIV risk for DMPA users increases (Figure 3B‐D). This is due to the assumption that HIV‐positive women are at greater risk of maternal death; under the hard change switching scenario, some of the women stopping DMPA use are HIV positive which puts them at increased risk of maternal death should they switch to no method and subsequently become pregnant. In the soft and medium change scenarios, only HIV‐negative women stop DMPA use, so this additional risk is avoided. If all women had the same MMR, the pattern of change in maternal deaths would replicate that of unsafe abortions.

### 3.4 DALYs averted

**Figure 4:**
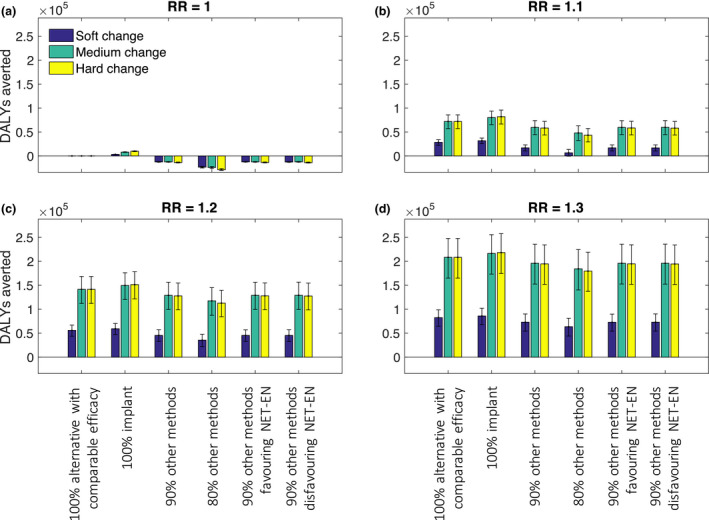
DALYs averted over 20 years under different assumed HRs for DMPA‐HIV risk association and different switching assumptions. Includes net HIV‐related and reproductive health DALYs. Uncertainty intervals represent 90% of variability in model outputs.

Figure 4 (and Table S7) summarize all the health outcomes in terms of DALYs averted in the whole population over 20 years under each contraceptive method mix change and DMPA risk assumption.

The change in DALYs strongly depends on the assumed HR. With no increased risk of HIV infection for DMPA users (HR = 1), there is no change in DALYs when all women switch to an alternative method of comparable effectiveness and no associated HIV risk (Figure 4A, replacement option 1) and a small positive health outcome (DALYs averted) when all women switch to the implant (Figure 4A, replacement 2). However, if 10% or 20% of DMPA users switch to no method then there is a net negative health outcome (more DALYs) over 20 years (Figure 4A, replacement 3 to 6).

In contrast, with an HR of 1.1 or higher, the benefits of a switch, in terms of reduced HIV risk, outweigh the increased adverse reproductive health outcomes, to give an overall increase in positive health outcomes (DALYs averted) over 20 years. This holds for all the switching scenarios – whether there is a complete replacement of contraceptive method for former DMPA users but even if 20% of former DMPA users move to no method. The net health gain is greatest when more women switch to an effective method. In all cases, the medium and hard change options (which see greater reductions in DMPA use) lead to more than twice the DALYs averted by a soft change in DMPA provision.

### 3.5 Value of a switching intervention

For the magnitude of health impact that could arise from a reduction in DMPA use, an intervention costing up to the order of $10M may be considered cost‐effective for the South African government as part of its HIV strategy (Table 3). For example under the medium change scenario, if there is a real but modest effect of DMPA on HIV risk such that HR = 1.2, then an intervention costing less than $54.7 million USD may be considered cost‐effective. But under HR = 1.1, an intervention to implement the medium change scenario would have to cost less than $2.9 million USD to be considered cost‐effective.

**Table 3:** Costs for a change in contraceptive usage consistent with being a marginally cost‐effective HIV intervention (USD, millions)

HR	Soft change	Medium change	Hard change
1.0^a^	n/a to 1.2	n/a to 2.9	n/a to 38.1
1.1	0.95 to 1.1	1.1 to 2.9	9.3 to 30.2
1.2	7.2 to 20.4	26.9 to 54.7	25.3 to 55.6
1.3	13.2 to 29.5	42.2 to 79.2	40.6 to 80.1

Total cost estimated by multiplying the range of projected life‐years saved (LYS), including 90% model variability interval, over 20 years under each assumed HR and switching scenario by the affordability range $547‐872/LYS.

^a^ If HR = 1.0, the overall outcome is harmful (loss of life‐years) when switching to a mix of existing methods and no method, therefore no cost is calculated; the outcome is neutral when all women switch to an alternative with comparable efficacy and no HIV risk; the outcome is beneficial when all women switch to the implant.

## 4 DISCUSSION

Following the ECHO trial, we can be confident that DMPA use does not confer a large additional HIV risk compared to the copper IUD or LNG implant. However, a Bayesian interpretation of all available evidence, including observational studies, may not rule out a smaller increase in HIV acquisition risk for women who use DMPA. It is therefore important for countries to consider the possible implications and the communication of this potential risk to women who are considering their contraceptive choices. This is especially true for countries such as South Africa where a high HIV incidence rate coincides with the high use of DMPA, and thus the impact of any true interaction would be largest. Conversely, countries with lower HIV incidence or DMPA prevalence would be affected less in absolute terms.

We find that if there was some undetected association between DMPA use and HIV acquisition risk – even with an HR as low as 1.1 – a situation where current DMPA users switch to another highly effective form of contraceptive could have a net health benefit over 20 years relative to the status quo. The South African government is arguably willing to pay up to the order of $10 million USD to secure this health gain, depending on the true size of any increased risk, based on other interventions that are currently funded as part of its HIV strategy.

The number of women that migrate away from DMPA and onto no method or a less effective method has the largest impact on adverse reproductive health outcomes. If all women moving away from DMPA can be efficiently transitioned onto a replacement method with similar or greater effectiveness, there are few if any harmful reproductive health consequences of reduced DMPA use. In contrast, if 20% of women on DMPA stop using contraception, there could be up to 106,000 extra unsafe abortions over 20 years with all other things being equal. Larger numbers of DMPA users switching to no contraceptive method could lead to a net reduction in population health.

We also find that a reduction in DMPA use among all women compared to only HIV‐negative women (“hard” vs. “medium” change) has no extra benefit in terms of the potential reduction in HIV infections, but does incur additional negative reproductive health outcomes, as maternal outcomes are worse for HIV‐positive women. This suggests that a tailored approach – focused on providing alternative contraceptive options to HIV‐negative women – may give the most optimal combined outcomes across different population groups.

A synthesis of evidence would require judgement on how to weight high‐quality evidence from a randomized trial against lower quality observational data. Furthermore, the different study types use different populations and different comparators. We therefore do not perform a formal synthesis here. We do note, however, that the high‐quality randomized data would likely dominate a combined effect measure, resulting in a central estimate closer to 1 than 1.3.

Balancing and comparing different forms of health outcomes, and especially for outcomes that have consequences beyond health, is not easy and relies to some extent on subjective assumptions. We have done this using DALYs. However, any decisions must weigh all considerations and in the light of locally accepted values and principles. One particular issue is the time‐frame for the analysis; although population behaviour and the available interventions may change substantially over 20 years, the long time‐frame is necessary for the HIV‐related outcomes to accrue (including long‐term ART use, AIDS deaths and indirect infections, which also affect men). Our long‐term, population‐wide view tries to take all these into account, whereas earlier work examining effects on women in the short‐term may reveal a more partial picture [27, 28, 29, 30, 31].

There are inherently a number of important assumptions that influence the results of this model. First, we have not incorporated the full range benefits of DMPA in particular, and contraception in general, for women to control the number and timing of the pregnancies and in a manner that is seemingly preferred by women in South Africa. We have also not considered the economic burden of unintended pregnancies, the adverse outcomes for children of reduced birth spacing and the increased risk of mother‐to‐child transmission of HIV.

The model assumes no behavioural differences between women choosing DMPA and women choosing other or no contraceptives. Such differences, if unadjusted for, may explain the discrepant results from the randomized trial and the observational studies, rather than simply lack of statistical power in the trial.

The model does not explicitly account for any increased HIV incidence during pregnancy or post‐partum. Although the physiological risk of HIV acquisition has been shown to be elevated for women during these periods [32], it remains unclear whether this translates into an increase in HIV incidence [33, 34]. If there was such an increase, the overall harms of women switching from DMPA to no method (or a less effective method) would be larger. Whether or not this effect would balance out any potential benefit of reduced HIV risk from DMPA use would depend on the true underlying HR and the pregnancy risk for women using no method or an alternative method.

We have assumed an interaction between HIV status (HIV‐positive women not on ART) and risk of maternal mortality that is supported by some but not all of the evidence [35]. We also had to rely on incomplete data to inform many parameters, especially those that affect the reproductive health outcomes. We have used national estimates where possible and regional estimates otherwise. We also used data on current patterns of contraceptive use to inform the model. However, method mix has been broadly stable for the past 20 years, and small variations will only have a modest effect on future projections of reproductive and HIV outcomes.

Finally, we have had to form assumptions about the nature of HIV programmes in the future. If we have over‐estimated future HIV incidence and under‐estimated the future success of ART programmes, the benefits in HIV outcomes from a reduced use of DMPA will have been over‐estimated. ART programs are set to grow and new initiatives such as the roll‐out of PrEP (which was not included in this model) could well see such positive changes unfold. In such cases, the benefits of a switch away from DMPA in reducing HIV outcomes are reduced and may be exceeded by the concomitant increase in adverse reproductive health outcomes.

## 5 CONCLUSIONS

Many countries should consider carefully the implications of the ECHO trial results. Both no relationship and a 30% increase in HIV risk for women using DMPA could be consistent with the totality of data, although the high‐quality randomized evidence points towards no association. Nevertheless, the possibility that HR > 1 cannot be excluded and, if true, would result in women using DMPA being subject to an excess HIV risk. Our analysis highlights and attempts to quantify the potential repercussions of the uncertainty around this complex issue. Accurate communication of risk and uncertainty is extremely difficult, especially in circumstances when the strength of evidence is open to different interpretations. Nevertheless, this uncertainty should be acknowledged and communicated to women so that they may make their own contraceptive choices. Ultimately, the only way to minimize even the potential for overall harm is to continue to focus on broadening the contraceptive method mix to provide safe, effective and acceptable alternatives to DMPA.

## COMPETING INTERESTS

Dr Smith reports grants from USAID and the Bill and Melinda Gates Foundation during the conduct of the study; and personal fees from the Bill and Melinda Gates Foundation outside the submitted work. Mr Beacroft reports grants from USAID and the Bill and Melinda Gates Foundation during the conduct of the study; personal fees from the Bill and Melinda Gates Foundation outside the submitted work; and travel expenses from WHO outside the submitted work. Prof Hallett reports grants USAID and the Bill and Melinda Gates Foundation during the conduct of the study; grants from BMGF, World Bank, UNAIDS, Rush Foundation and Wellcome Trust; and personal fees from BMGF, New York University, WHO and GFATM outside the submitted work.

## AUTHORS’ CONTRIBUTIONS

JAS, LB and TBH designed the preliminary analysis. JAS produced the preliminary results. Upon consultation with FA, BB, MM, CM, GR and CV, the analysis plan was revised. JAS produced the revised results. JAS, LB, FA, BB, MM, CM, GR, CV and TBH wrote the manuscript.

## Supporting information


**Figure S1.** Natural history of HIV infection and ART initiation as represented in the model.
Movement between compartments is indicated by arrows.
**Figure S2.** The proportion of adult men that are circumcised with respect to time.
The level of circumcision in the model was calibrated to data reported in a nationally representative survey.
**Figure S3.** The number of adults receiving antiretroviral therapy in South Africa. Model data is compared to estimates of the number of adults on ART in South Africa. Grey lines represent runs of the model using different parameter sets.
**Figure S4.** Maternal mortality. The model was calibrated to estimates of MMR from the Institute of Health Metrics as well as estimates from the South African Rapid Mortality Surveillance. Grey lines represent model runs using different parameter sets.
**Figure S5.** Population pyramids for South Africa for 1985, 1990, 1995, 2000, 2005 and 2010. Model population structure is compared to annual age‐structured population size model estimates produced by the Actuarial Society of South Africa.
**Figure S6.** Population size with respect to time. The total population of the model was calibrated to previous model estimates.
**Figure S7.** Model calibration to HIV prevalence data
The model was calibrated adult HIV prevalence data from a nationally representative survey and previous estimates of HIV prevalence. The grey lines represent different model parameter sets.
**Figure S8.** Model calibration to HIV incidence data. The model was calibrated adult HIV incidence data from a nationally representative survey as well as previous incidence estimates from a mathematical model. The grey lines represent different model parameter sets.
**Figure S9.** Model calibration to male HIV prevalence data. The model was calibrated adult male HIV prevalence data from a nationally representative survey. The grey lines represent different model parameter sets.
**Figure S10.** Model calibration to female HIV prevalence data. The model was calibrated adult female HIV prevalence data from a nationally representative survey. The grey lines represent different model parameter sets.
**Figure S11.** HIV prevalence by age group (men). HIV prevalence in the model was calibrated to sex and age‐specific prevalence data (a single‐year example calibration is shown here for clarity).
**Figure S12.** HIV prevalence by age group (women). HIV prevalence in the model was calibrated to sex and age‐specific prevalence data (a single‐year example calibration is shown here for clarity).
**Figure S13.** Change in HIV infections among women over five years under different assumed HRs for DMPA‐HIV risk association and different switching assumptions. Uncertainty intervals represent 90% of variability in model outputs.
**Figure S14.** Change in HIV infections among women over ten years under different assumed HRs for DMPA‐HIV risk association and different switching assumptions. Uncertainty intervals represent 90% of variability in model outputs.
**Figure S15.** Change in unsafe abortions over five years under different assumed HRs for DMPA‐HIV risk association and different switching assumptions. Uncertainty intervals represent 90% of variability in model outputs.
**Figure S16.** Change in unsafe abortions over ten years under different assumed HRs for DMPA‐HIV risk association and different switching assumptions. Uncertainty intervals represent 90% of variability in model outputs.
**Figure S17.** DALYs averted over five years under different assumed HRs for DMPA‐HIV risk association and different switching assumptions.Uncertainty intervals represent 90% of variability in model outputs.
**Figure S18.** DALYs averted over ten years under different assumed HRs for DMPA‐HIV risk association and different switching assumptions. Uncertainty intervals represent 90% of variability in model outputs.

**Table S1.** Natural history of infection parameters
**Table S2.** Behavioural parameters and values
**Table S3.** Factors affecting transmission probability per sex act with respect to baseline transmission probability (β_0_)
**Table S4.** Contraceptive efficacy and continuation rates for methods used in the model
**Table S5.** Parameters for reproductive health outcomes
**Table S6.** Calibrated parameters
**Table S7.** Total DALYs averted over 20 years (thousands)
