# Supplementary material for: Responding to the ECHO trial results: modelling the potential impact of changing contraceptive method mix on HIV and reproductive health in South Africa
Source: J Int AIDS Soc. 2020 Oct 8;23(10):e25620. doi: 10.1002/jia2.25620 (PMC7543057; doi:10.1002/jia2.25620)
Supplement: Supplementary file 1 — Figure S1. Natural history of HIV infection and ART initiation as represented in the model. Movement between compartments is indicated by arrows. Figure S2. The proportion of adult men that are circumcised with respect to time. The level of circumcision in the model was calibrated to data reported in a nationally representative survey. Figure S3. The number of adults receiving antiretroviral therapy in South Africa. Model data is compared to estimates of the number of adults on ART in South Africa. Grey lines represent runs of the model using different parameter sets. Figure S4. Maternal mortality. The model was calibrated to estimates of MMR from the Institute of Health Metrics as well as estimates from the South African Rapid Mortality Surveillance. Grey lines represent model runs using different parameter sets. Figure S5. Population pyramids for South Africa for 1985, 1990, 1995, 2000, 2005 and 2010. Model population structure is compared to annual age‐structured population size model estimates produced by the Actuarial Society of South Africa. Figure S6. Population size with respect to time. The total population of the model was calibrated to previous model estimates. Figure S7. Model calibration to HIV prevalence data The model was calibrated adult HIV prevalence data from a nationally representative survey and previous estimates of HIV prevalence. The grey lines represent different model parameter sets. Figure S8. Model calibration to HIV incidence data. The model was calibrated adult HIV incidence data from a nationally representative survey as well as previous incidence estimates from a mathematical model. The grey lines represent different model parameter sets. Figure S9. Model calibration to male HIV prevalence data. The model was calibrated adult male HIV prevalence data from a nationally representative survey. The grey lines represent different model parameter sets. Figure S10. Model calibration to female HIV prevalence data. The model was calibrated adu [file JIA2-23-e25620-s001.docx]

Preparing for the ECHO Trial Results: Modelling the Potential Impact of Changing Contraceptive Method Mix on HIV and Reproductive Health in South Africa

Supporting Information – Figures

**Figure S1. Natural history of HIV infection and ART initiation as represented in the model.**

Movement between compartments is indicated by arrows.


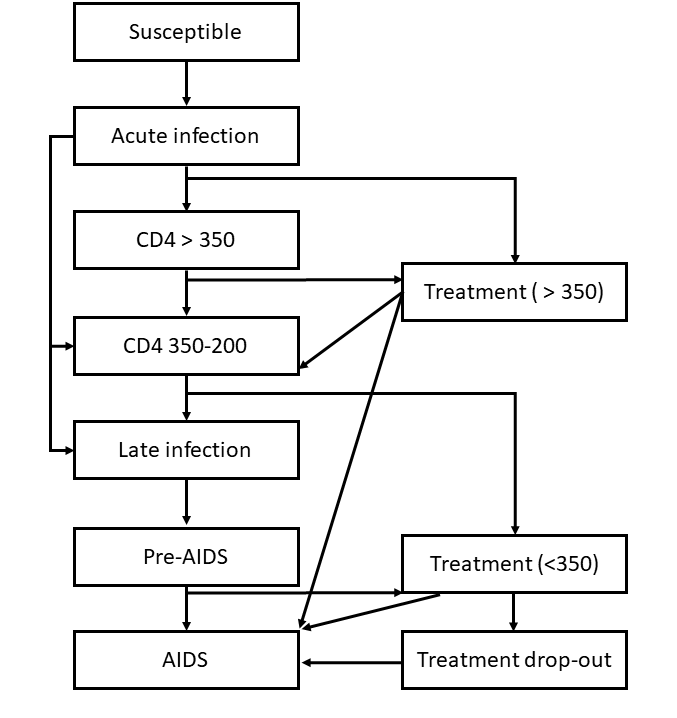


**Figure S2. The proportion of adult men that are circumcised with respect to time.**

**The level of circumcision in the model was calibrated to data reported in a nationally representative survey.^1^**


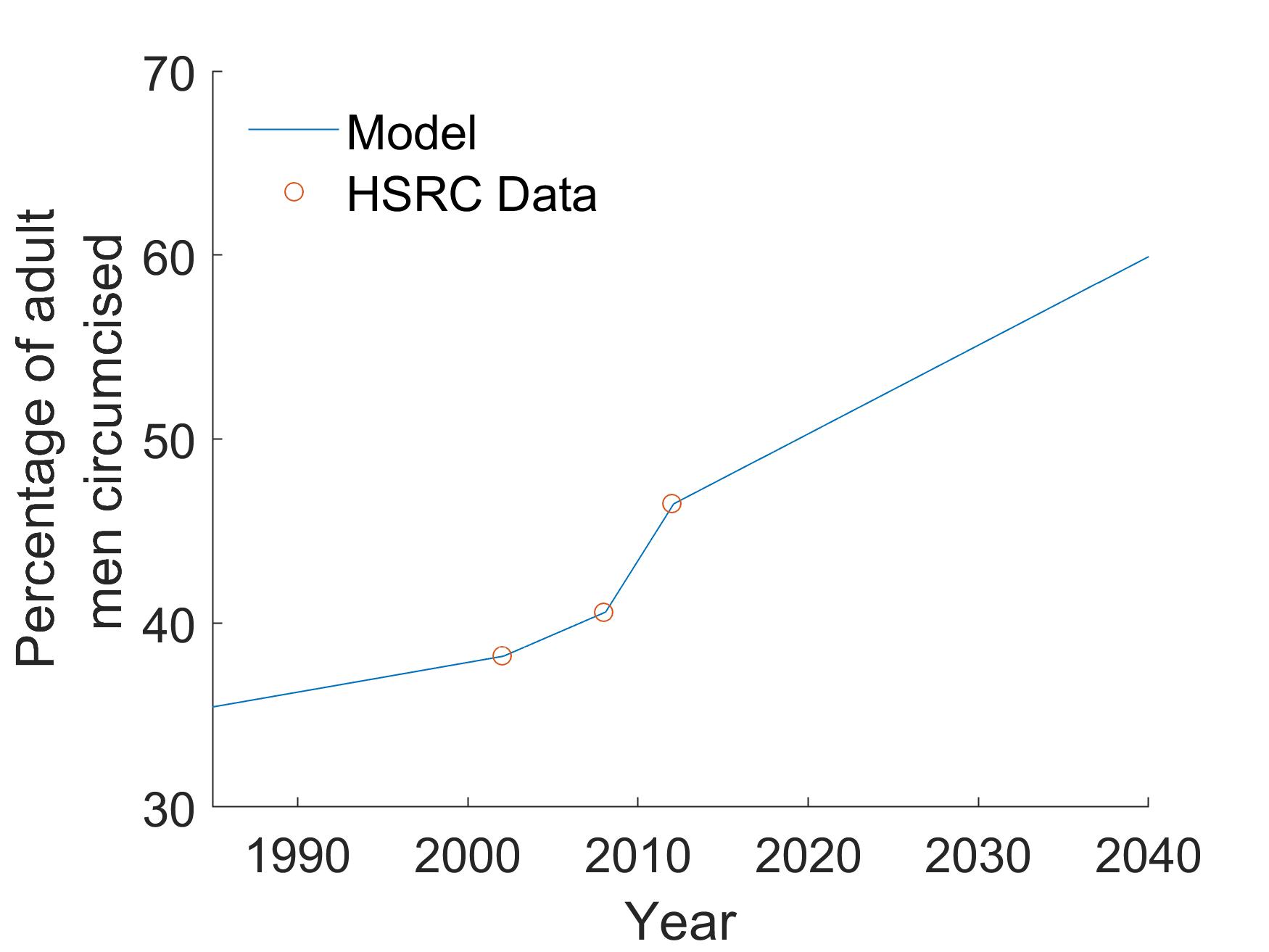


**Figure S3. The number of adults receiving antiretroviral therapy in South Africa.**

Model data is compared to estimates of the number of adults on ART in South Africa.^2,3^ Grey lines represent runs of the model using different parameter sets.


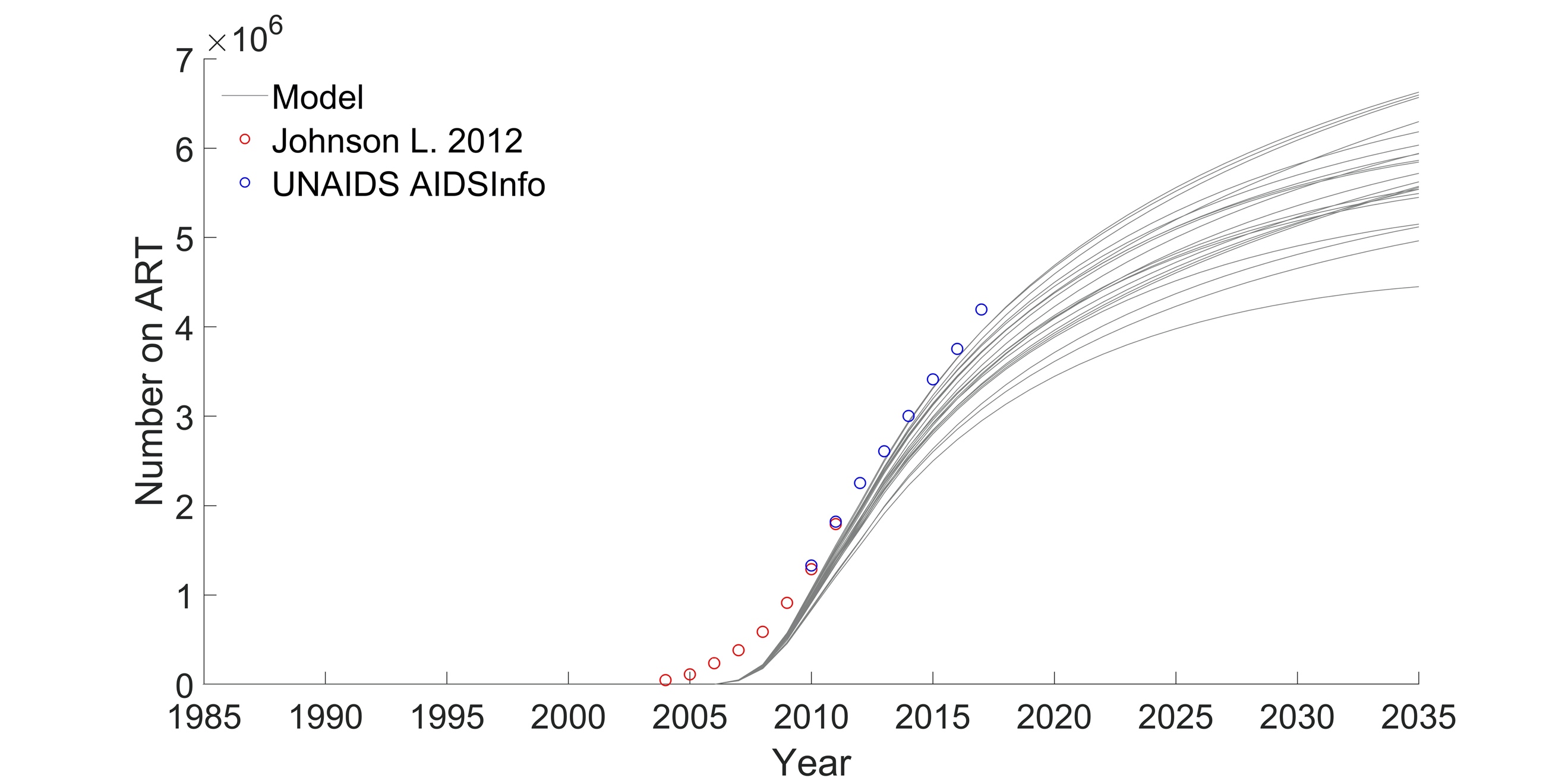


**Figure S4. Maternal mortality**

The model was calibrated to estimates of MMR from the Institute of Health Metrics as well as estimates from the South African Rapid Mortality Surveillance.^4-7^ Grey lines represent model runs using different parameter sets.


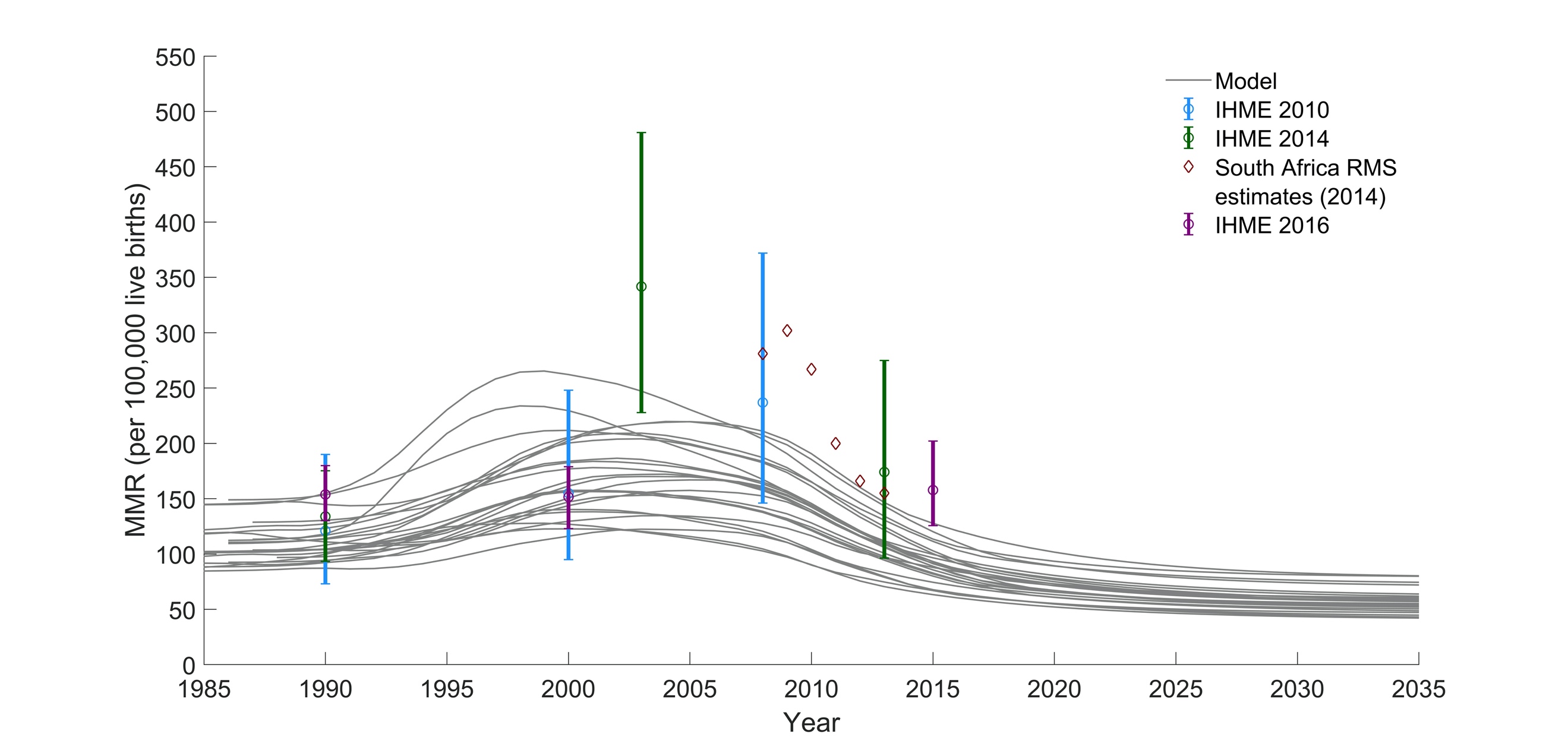


**Figure S5. Population pyramids for South Africa for 1985, 1990, 1995, 2000, 2005 and 2010.** Model population structure is compared to annual age-structured population size model estimates produced by the Actuarial Society of South Africa.^8^


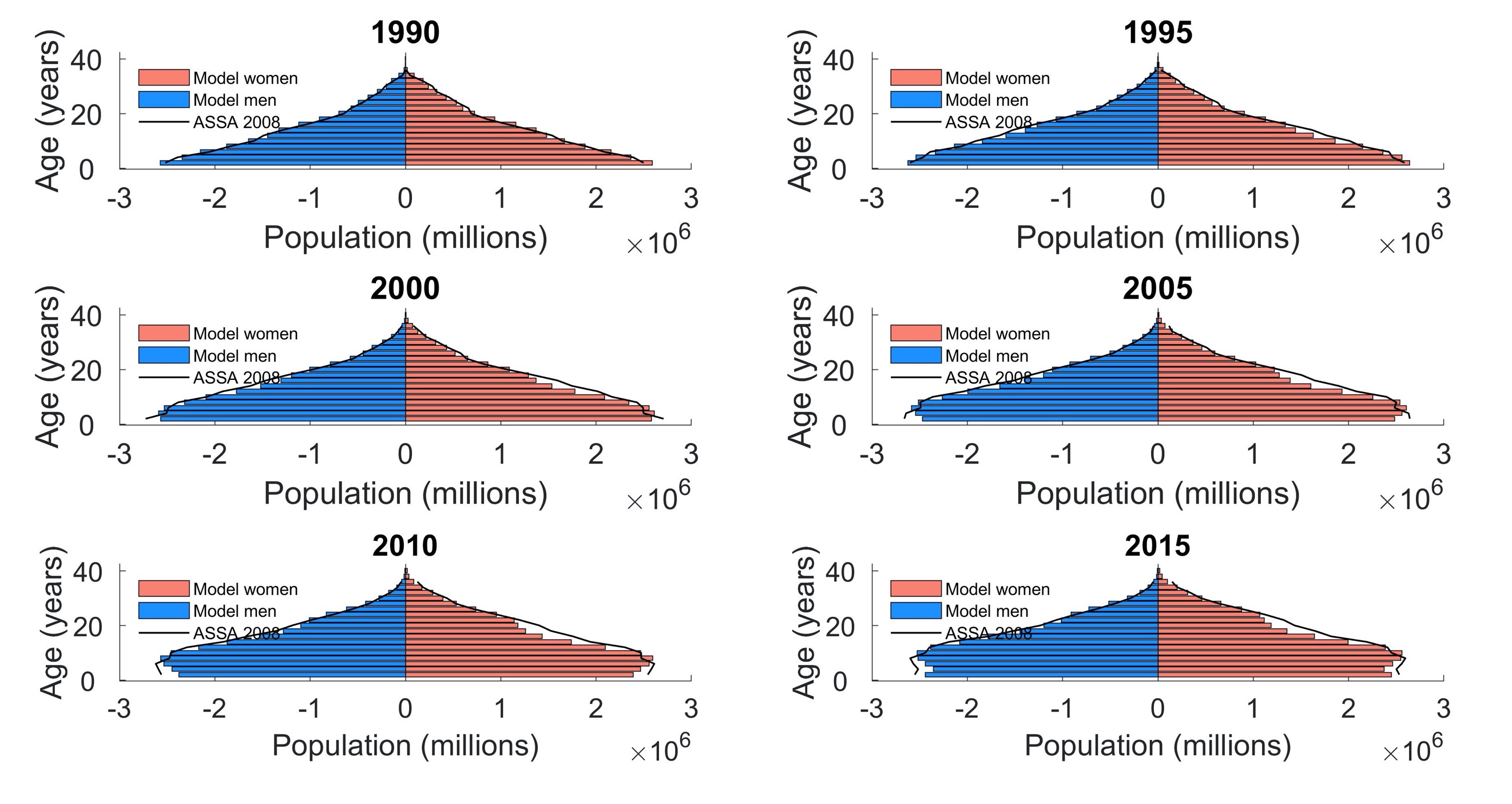


**Figure S6. Population size with respect to time.**

The total population of the model was calibrated to previous model estimates.^8^


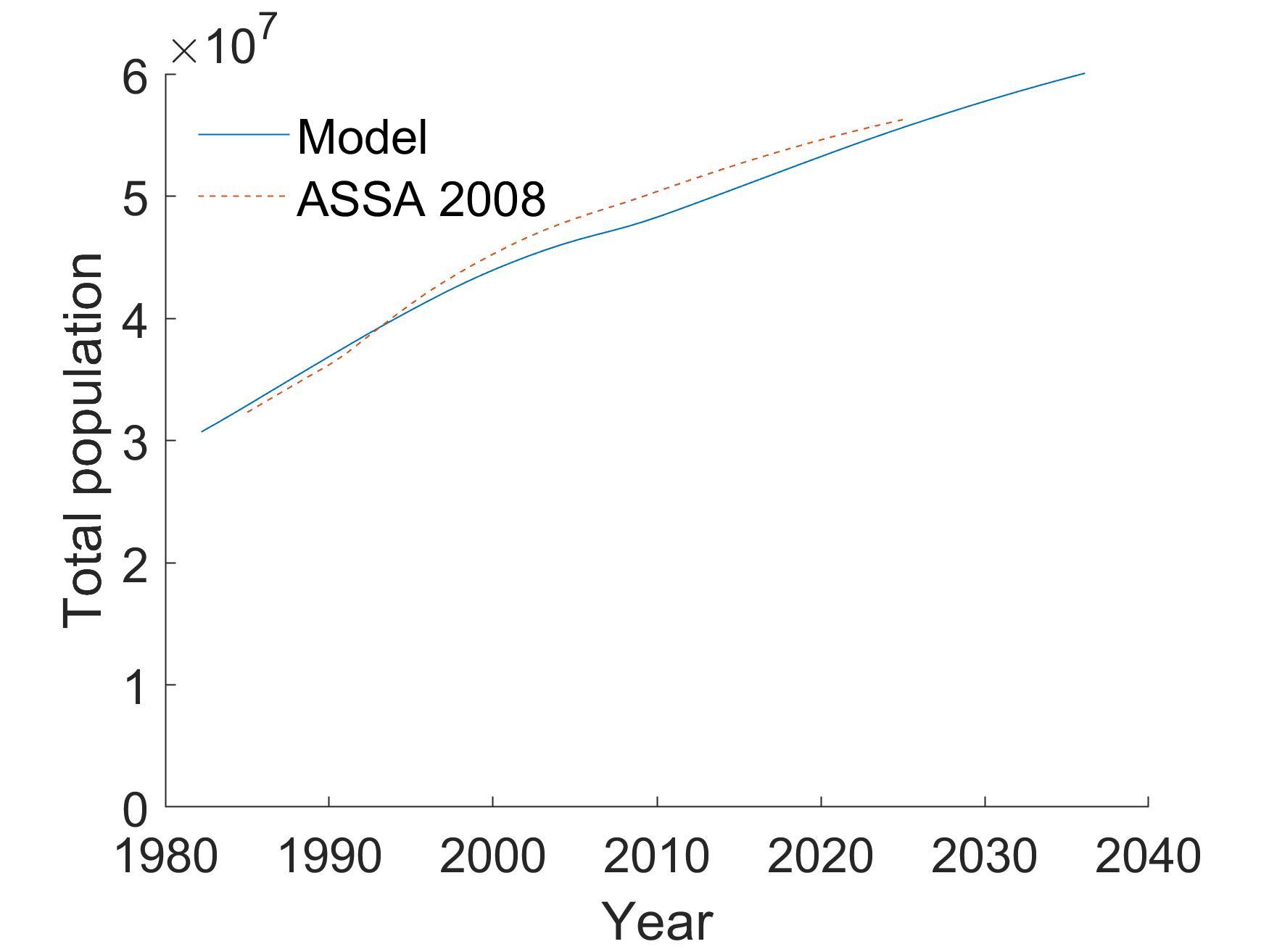


**Figure S7. Model calibration to HIV prevalence data**

The model was calibrated adult HIV prevalence data from a nationally representative survey and previous estimates of HIV prevalence.^1,9^ The grey lines represent different model parameter sets.


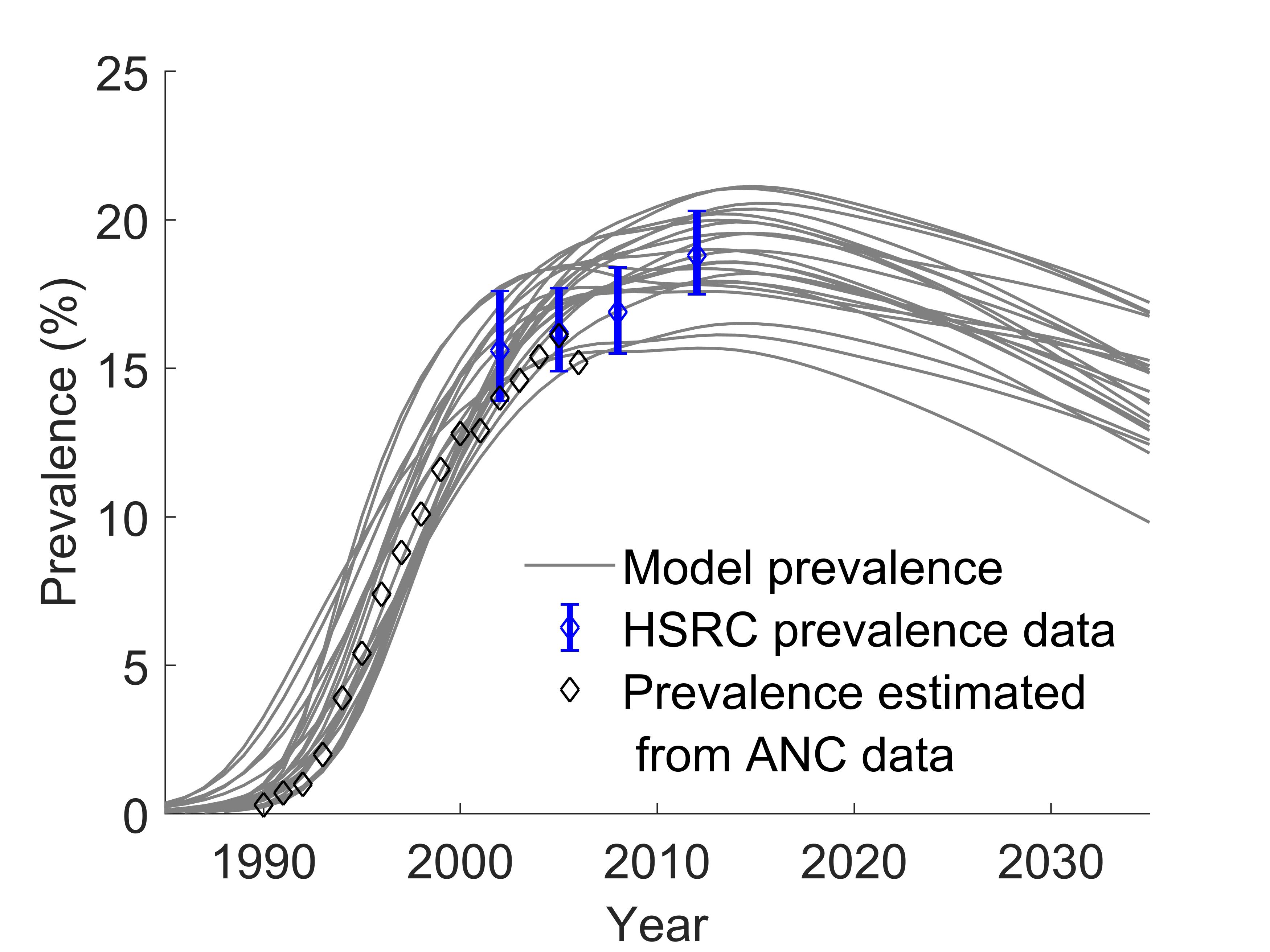


**Figure S8. Model calibration to HIV incidence data**

The model was calibrated adult HIV incidence data from a nationally representative survey as well as previous incidence estimates from a mathematical model.^1^ The grey lines represent different model parameter sets.


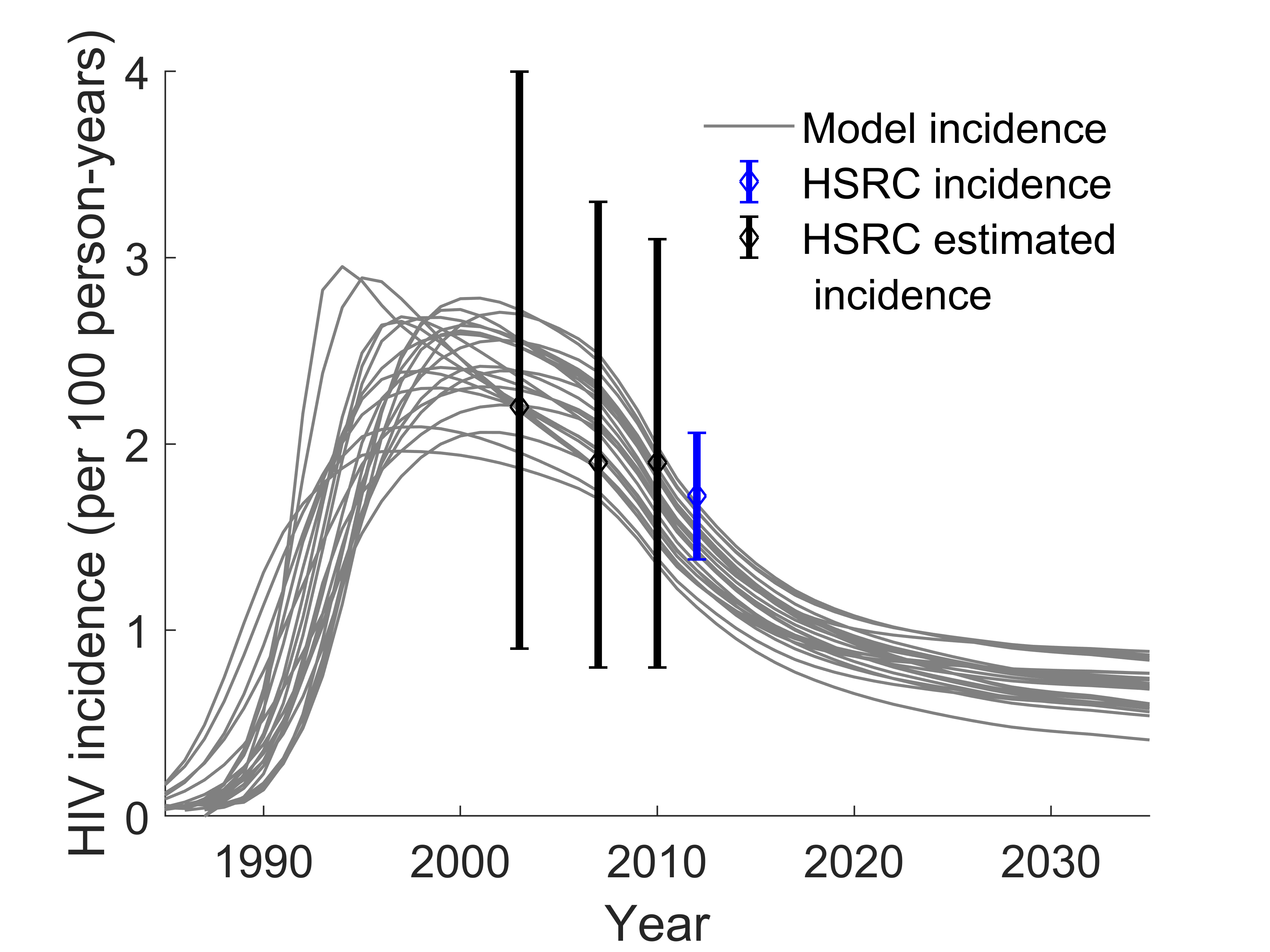


**Figure S9. Model calibration to male HIV prevalence data**

The model was calibrated adult male HIV prevalence data from a nationally representative survey.^1^ The grey lines represent different model parameter sets.


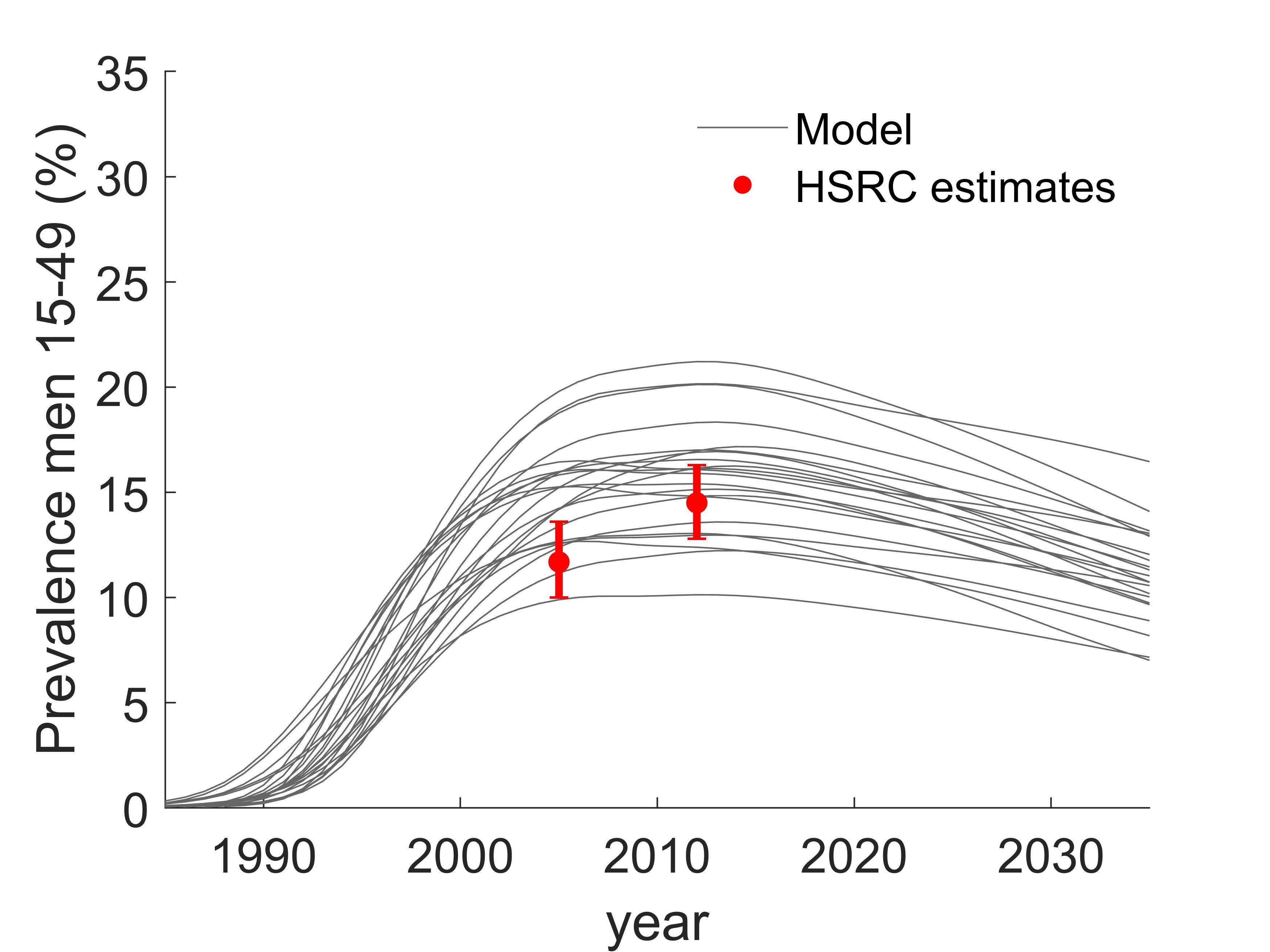


**Figure S10. Model calibration to female HIV prevalence data**

The model was calibrated adult female HIV prevalence data from a nationally representative survey.^1^ The grey lines represent different model parameter sets.


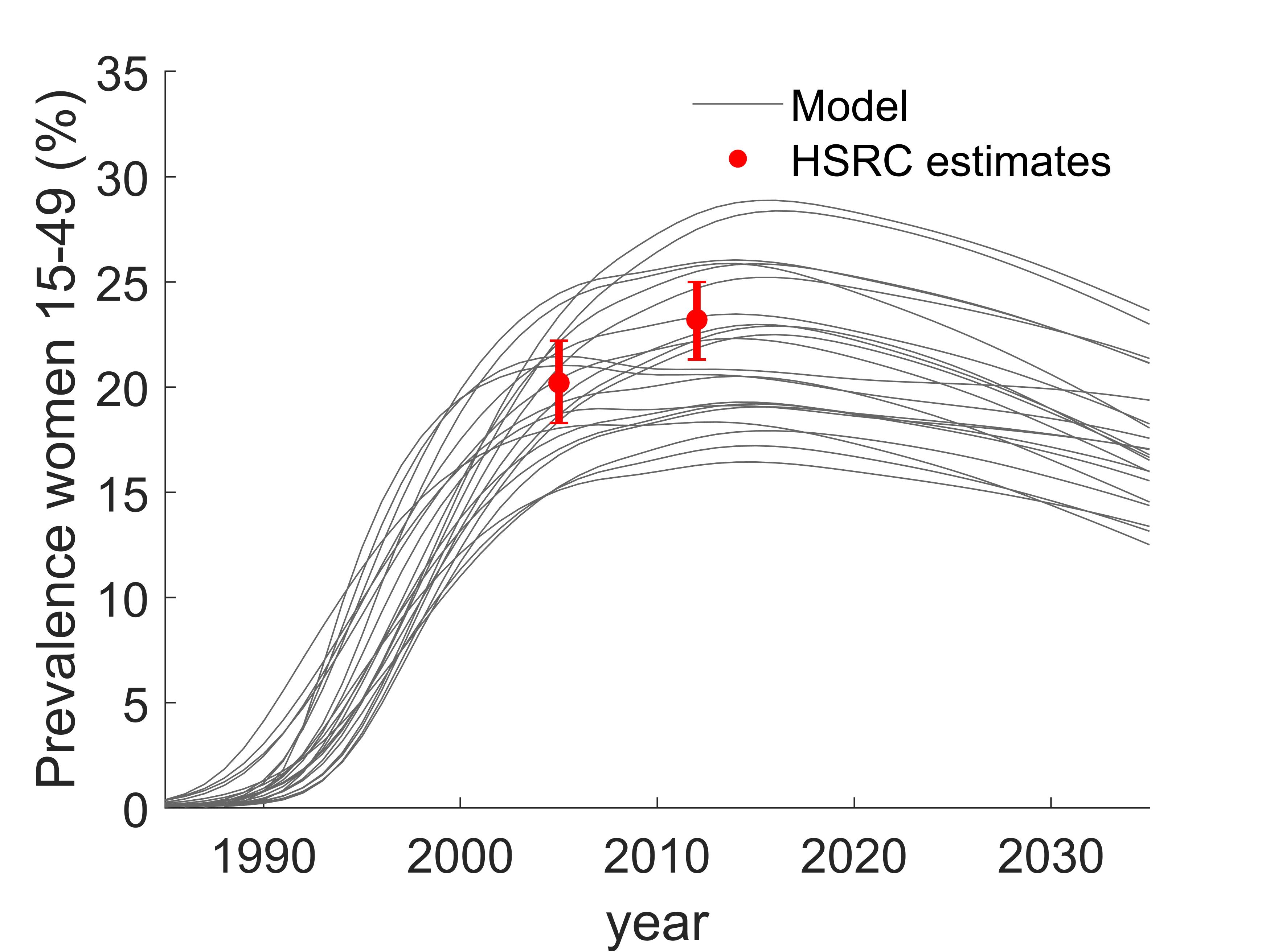


**Figure S11. HIV prevalence by age group (men)**

HIV prevalence in the model was calibrated to sex and age-specific prevalence data (a single-year example calibration is shown here for clarity).^1^

**Figure S12. HIV prevalence by age group (women)**

HIV prevalence in the model was calibrated to sex and age-specific prevalence data (a single-year example calibration is shown here for clarity).^1^

**Figure S13. Change in HIV infections among women over five years under different assumed HRs for DMPA-HIV risk association and different switching assumptions.**
Uncertainty intervals represent 90% of variability in model outputs.


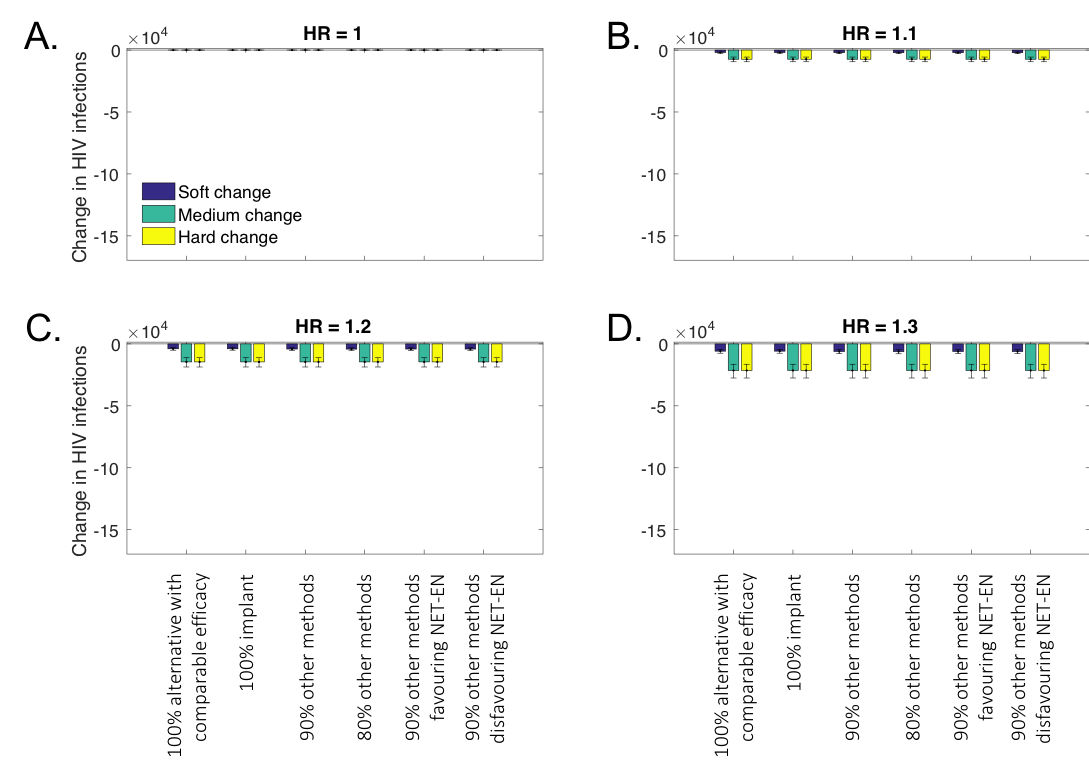


**Figure S14. Change in HIV infections among women over ten years under different assumed HRs for DMPA-HIV risk association and different switching assumptions.**
Uncertainty intervals represent 90% of variability in model outputs.


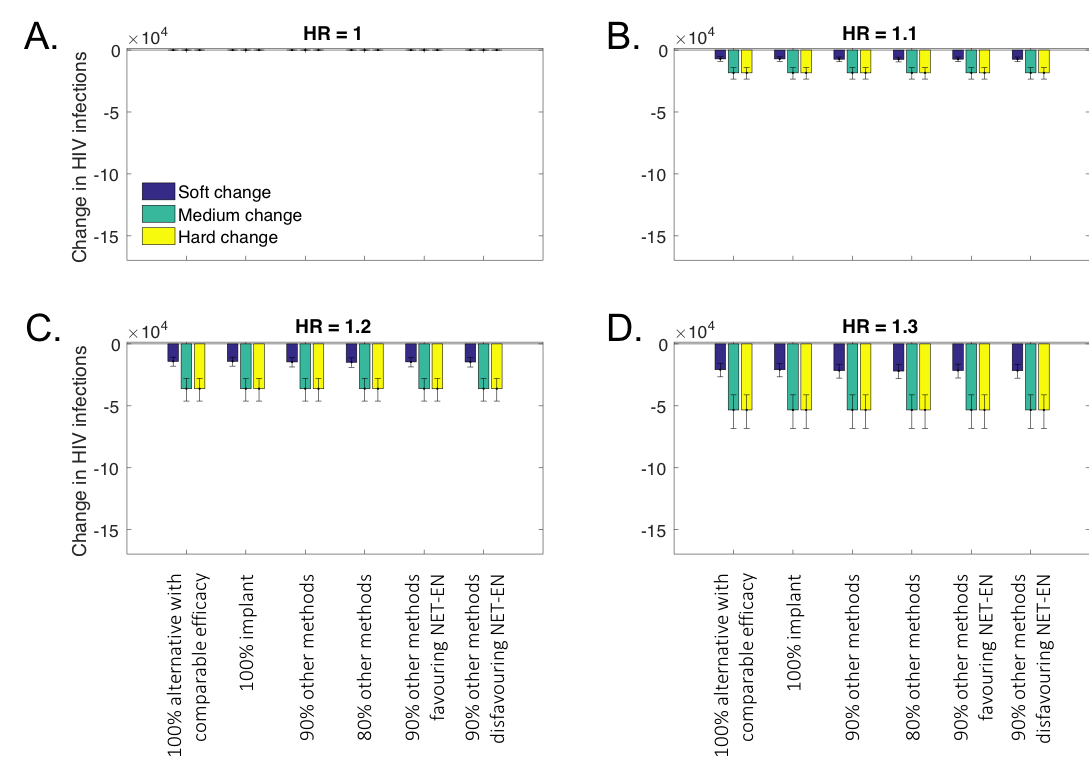


**Figure S15. Change in unsafe abortions over five years under different assumed HRs for DMPA-HIV risk association and different switching assumptions.**
Uncertainty intervals represent 90% of variability in model outputs.


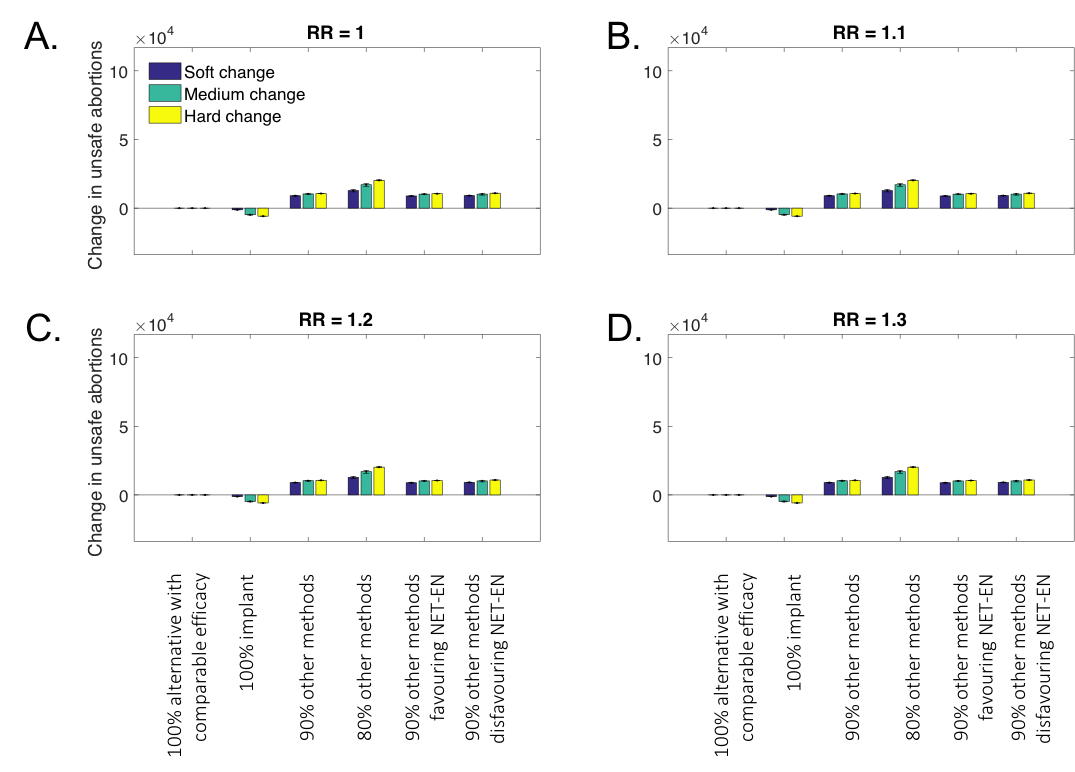


**Figure S16. Change in unsafe abortions over ten years under different assumed HRs for DMPA-HIV risk association and different switching assumptions.**
Uncertainty intervals represent 90% of variability in model outputs.


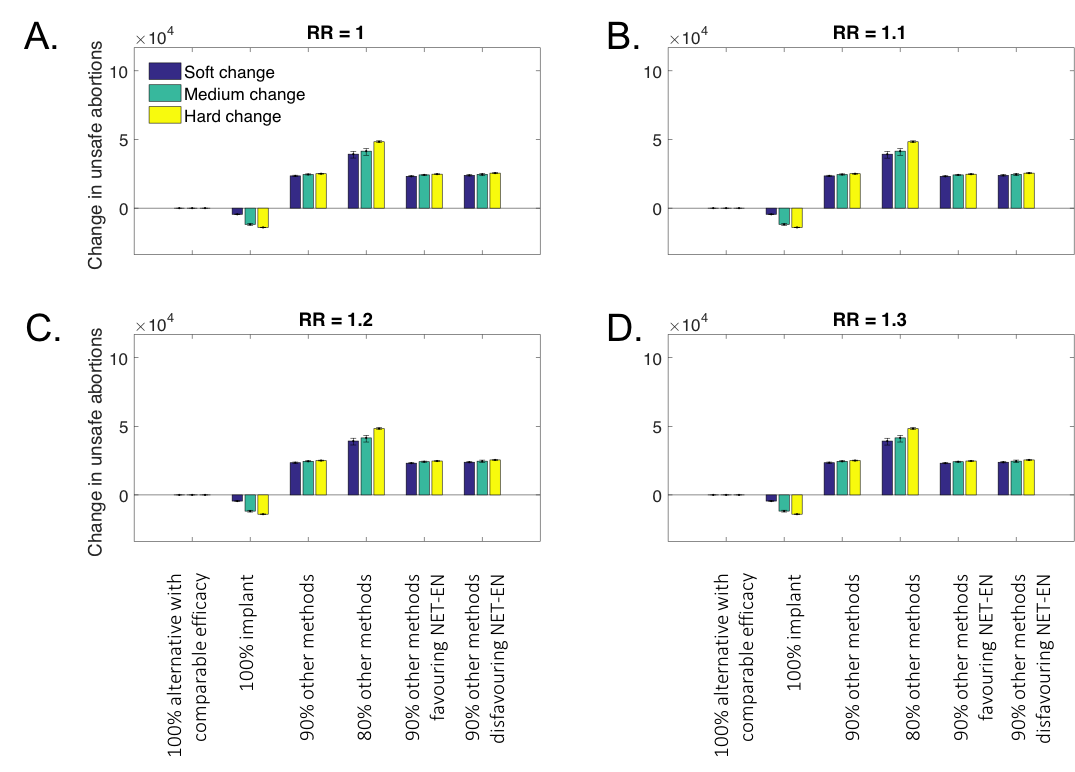


**Figure S17. DALYs averted over five years under different assumed HRs for DMPA-HIV risk association and different switching assumptions.**
Uncertainty intervals represent 90% of variability in model outputs.


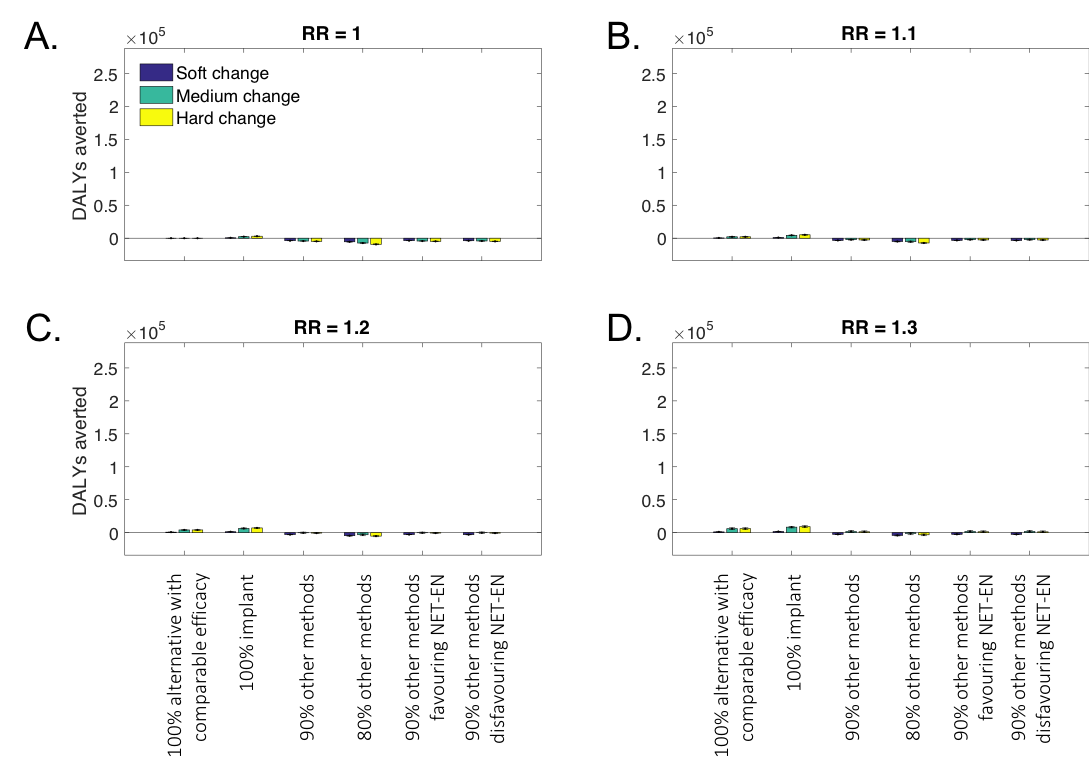


**Figure S18. DALYs averted over ten years under different assumed HRs for DMPA-HIV risk association and different switching assumptions.**Uncertainty intervals represent 90% of variability in model outputs.


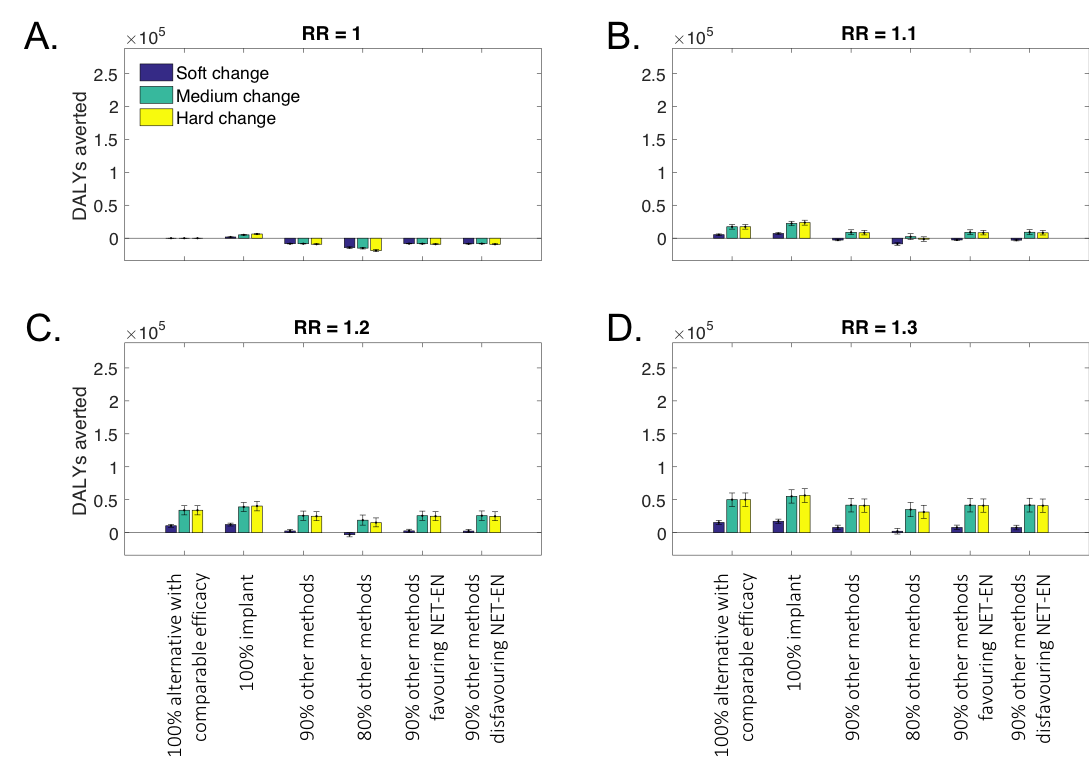


**References**

1. Shisana O, Rehle T, Simbayi LC, et al. South African National HIV Prevalence, Incidence and Behaviour Survey, 2012. Cape Town, 2014.

2. Johnson LF. Access to antiretroviral treatment in South Africa, 2004 - 2011. *2012* 2012; **13**(1): 6.

3. UNAIDS. AIDSInfo. 2018. aidsinfo.unaids.org (accessed 12th February 2019).

4. Kassebaum NJ, Barber RM, Bhutta ZA, et al. Global, regional, and national levels of maternal mortality, 1990-2015: a systematic analysis for the Global Burden of Disease Study 2015. *The Lancet* 2016; **388**(10053): 1775-812.

5. Kassebaum NJ, Bertozzi-Villa A, Coggeshall MS, et al. Global, regional, and national levels and causes of maternal mortality during 1990-2013: a systematic analysis for the Global Burden of Disease Study 2013. *The Lancet* 2014.

6. Hogan MC, Foreman KJ, Naghavi M, et al. Maternal mortality for 181 countries, 1980-2008: a systematic analysis of progress towards Millennium Development Goal 5. *The Lancet* 2010; **375**(9726): 1609-23.

7. Dorrington R, Bradshaw D, Laubscher R, Nannan N. Rapid mortality surveillance report 2016. Cape Town: South African Medical Research Council; 2018.

8. Dorrington R, Johnson L, Budlender D. ASSA2008 AIDS and Demographic Models User Guise (beta version). Cape Town: Centre for Actuarial Research, University of Cape Town, 2010.

9. Granich RM, Gilks CF, Dye C, De Cock KM, Williams BG. Universal voluntary HIV testing with immediate antiretroviral therapy as a strategy for elimination of HIV transmission: a mathematical model. *The Lancet* 2009; **373**(9657): 48-57.
