# Supplementary material for: Responding to the ECHO trial results: modelling the potential impact of changing contraceptive method mix on HIV and reproductive health in South Africa
Source: J Int AIDS Soc. 2020 Oct 8;23(10):e25620. doi: 10.1002/jia2.25620 (PMC7543057; doi:10.1002/jia2.25620)
Supplement: Supplementary file 2 — Table S1. Natural history of infection parameters Table S2. Behavioural parameters and values Table S3. Factors affecting transmission probability per sex act with respect to baseline transmission probability (β0) Table S4. Contraceptive efficacy and continuation rates for methods used in the model Table S5. Parameters for reproductive health outcomes Table S6. Calibrated parameters Table S7. Total DALYs averted over 20 years (thousands) [file JIA2-23-e25620-s002.docx]

Preparing for the ECHO Trial Results: Modelling the Potential Impact of Changing Contraceptive Method Mix on HIV and Reproductive Health in South Africa

Supporting Information

1. **South Africa Epidemic Model**

# 1.1 Model overview

The model, adapted from a model developed by Cremin and co-authors,[^1-3^](#_ENREF_1) is a deterministic compartmental model defined by a set of ordinary differential equations. It is designed to represent heterosexual HIV transmission at the population level in South Africa, a mature, generalised HIV epidemic, and to estimate the effect of changes in contraceptive use in terms of HIV and reproductive health outcomes. Our aim is to estimate how these outcomes may be affected by changes in contraceptive policy in response to the results of the ECHO trial.

The model population is divided into compartments that are distinguished by sex, circumcision status (if male), age, infection stage, sexual behaviour and contraceptive use, with events (e.g. HIV infection, death, ART initiation etc.) represented as movement between these compartments.[^4-6^](#_ENREF_4) Heterogeneity in sexual behaviour is incorporated in the model by stratifying men and women into three risk groups according to their average effective partnership formation rate.

A full description of the model structure, parameter values used and calibration is provided under the following sections: 1.2 Natural History of HIV infection, 1.3 Demography, 1.4 HIV transmission and sexual mixing, 1.5 Male circumcision, 1.6 Antiretroviral treatment, 1.7 Contraception, 1.8 Reproductive health outcomes, 1.9 Model calibration.

# 1.2 Natural history of HIV infection

A flow diagram for the natural history of HIV infection and initiation on ART is shown in Figure S1. The model is specified by the following ordinary differential equations:

The equations describing susceptible individuals are:

$\frac{dX_{l,k,1}^{0,p}}{\mathrm{dt}}=\mu N\Psi_{\left( l,k,p,1 \right)}-\left( \lambda_{l,k,1}^{p}+\mu_{k,1} \right)X_{l,k,1}^{0,p}+f\left( 1 \right)+g(k)+ q(p)$ for a=1

$\frac{dX_{l,k,a}^{0,p}}{\mathrm{dt}}=-\left( \lambda_{l,k,A}^{p}+\mu_{k,a} \right)X_{l,k,a}^{0,p}+f\left( a \right)+g(k)+ q(p)$ for a≠1

(1)

The equations describing HIV infected individuals are:

$$\frac{dX_{l,k,a}^{1,p}}{\mathrm{dt}}=\lambda_{l,k,A}^{p}X_{l,k,a}^{0,p}-\left( {(\gamma1+\gamma2+\gamma3)\sigma}_{1}+\mu_{k,a} \right)X_{l,k,a}^{1,p}+f\left( a \right) + g(k)+ q(p)$$

$$\frac{dX_{l,k,a}^{2,p}}{\mathrm{dt}}=\left( 1-\phi_{1} \right)\sigma_{1}{\gamma1X}_{l,k,a}^{1,p}+\zeta_{E}X_{l,k,a}^{7,p}-\left( \sigma_{2}+\mu_{k,a} \right)X_{l,k,a}^{2,p}+f\left( a \right) + g(k)+ q(p)$$

$$\frac{dX_{l,k,a}^{3,p}}{\mathrm{dt}}=\left( 1-\phi_{2} \right)\sigma_{2}\gamma2X_{l,k,a}^{2,p}-\left( \sigma_{3}+\mu_{k,a} \right)X_{l,k,a}^{3,p}+f\left( a \right)+ g(k)+ q(p)$$

$$\frac{dX_{l,k,a}^{4,p}}{\mathrm{dt}}=\left( 1-\phi_{3} \right)\sigma_{3}\gamma3X_{l,k,a}^{3,p}-\left( \sigma_{4}+\mu_{k,a} \right)X_{l,k,a}^{4,p}+f\left( a \right)+ g(k)+ q(p)$$

$$\frac{dX_{l,k,a}^{5,p}}{\mathrm{dt}}=\sigma_{4}X_{l,k,a}^{4,p}-\left( \sigma_{5}+\mu_{k,a} \right)X_{l,k,a}^{5,p}+f\left( a \right)+ g(k)+ q(p)$$

$$\frac{dX_{l,k,a}^{6,p}}{\mathrm{dt}}=\left( 1-\phi_{4} \right)\sigma_{5}X_{l,k,a}^{5,p}+\omega_{E}X_{l,k,a}^{7,p}+\omega_{L}X_{l,k,a}^{8,p}+\tau_{D}X_{l,k,a}^{9,p}-\left( \Omega+\mu_{k,a} \right)X_{l,k,a}^{6,p}+f\left( a \right)\ldots+ g\left( k \right)+ q(p)$$

$$\frac{dX_{l,k,a}^{7,p}}{\mathrm{dt}}={\phi_{3}\sigma}_{3}X_{l,k,a}^{3,p}+{\phi_{4}\sigma}_{5}X_{l,k,a}^{5,p}-\left( \omega_{L}+\zeta_{L}+\mu_{k,a} \right)X_{l,k,a}^{7,p}+f\left( a \right)+ g(k)+ q(p)$$

$$\frac{dX_{l,k,a}^{8,p}}{\mathrm{dt}}={\phi_{1}\sigma}_{1}\gamma1X_{l,k,a}^{1,p}+{\phi_{2}\sigma}_{2}X_{l,k,a}^{2,p}-\left( \omega_{E}+\zeta_{E}+\mu_{k,a} \right)X_{l,k,a}^{8,p}+f\left( a \right)+ g(k)+ q(p)$$

$$\frac{dX_{l,k,a}^{9,p}}{\mathrm{dt}}=\zeta_{L}X_{l,k,a}^{8,p}-\left( \tau_{D}+\mu_{k,a} \right)X_{l,k,a}^{9,p}+f\left( a \right)+ g(k)+ q(p)$$

(2)

For a given stage of HIV infection, hazards of progression to the next stage are given by the rates σ _(1,2,3,4,5)_. The late infection stage is defined by the mean time between when CD4 count falls below 200 cells/μl and viremic rebound 19 months, on average, before death. The pre-AIDS stage characterizes the 9 month period of heightened infectiousness before AIDS, which represents a 10 month period of no transmission risk.[^7^](#_ENREF_7)

Mortality in the AIDS stage is denoted by the parameter Ω. An important limitation of this model is that AIDS-related mortality only applies to this final AIDS stage. However, in reality some infected individuals may die of AIDS-related illnesses at higher CD4 counts.

Several representations of ART initiation are possible in the model; ART can be initiated following acute infection, when an individual’s CD4 count drops below 350, 200, or 100 cells per microliter. The proportion of individuals initiating ART following acute infection, at CD4 <350 cells/μl, CD4 <200 cells/μl and CD4 <100 cells/μl are controlled by the parameters ϕ_1,_ ϕ_2_, ϕ_3_ and ϕ_4,_ respectively_._  ART initiation at low CD4 counts (< 100 cells/μl) is used to represent the initial pattern of ART initiation (ie. for urgent clinical need), when ART was first introduced in South Africa.

ART is assumed to extend the survival of treated individuals (the increase in life expectancy depends on whether ART is initiated ‘≥350 cells/μl’ or ‘<350 cells/μl’) while reducing infectiousness.[^8^](#_ENREF_8)^,^[^9^](#_ENREF_9) Individuals initiating ART ≥350 cells/μl or <350 cells/μl are assumed to survive on average $\frac{1}{\omega_{E}}$ or $\frac{1}{\omega_{L}}$ years before progressing to AIDS, respectively. Drop outs from treatment initiated ≥350 cells/μl return to having a CD4 ≥350 cells/μl and progress through infection. Drop outs from treatment when initiated <350 cells/μl’ progress to AIDS after a period of slightly heightened infectiousness represented by the ‘Treatment drop-out’ compartment in Figure S1.

**Table S1: Natural history of infection parameters**

| Parameter | Symbol | Value | Source |
| --- | --- | --- | --- |
| Mean duration of acute infection | 1/σ_1_ | 0.25 years | [^7^](#_ENREF_7) |
| Mean duration from the end of acute infection to CD4 350 cells/μl | 1/σ_2_ | 9.25 years |  |
| Mean duration from CD4 350 cells/μl to CD4 200 cells/μl | 1/σ_3_ | 3.54 years |  |
| Mean duration from CD4<200 cells/μl to viremic rebound | 1/σ_4_ | 1.12 years |  |
| Mean duration of viremic rebound before AIDS | 1/σ_5_ | 0.75 years |  |
| AIDS mortality rate | Ω | 1/0.833 (10 month period before death) |  |
| Proportion of HIV infected individuals starting infection at CD4> 350 cells/μl | $\gamma1$ | 0.81  (0.58+0.23) | [^10^](#_ENREF_10) |
| Proportion of HIV infected individuals starting infection at CD4 200-350 cells/μl | $\gamma2$ | 0.16 |  |
| Proportion of HIV infected individuals starting infection at CD4<200 cells/μl | $\gamma3$ | 0.03 |  |

# 1.3 Demography

The model is stratified by one-tenth of a single year of age from birth to 100 years. Ageing of individuals is represented by:

$$X_{l,k,1}^{0,p}= X_{l,1,a}^{s,p}b_{a} for a=1$$

$$X_{l,k,a}^{s,p}=X_{l,k,a-1}^{s,p} for a>1$$

(3)

$\Psi_{\left( l,k,p,a \right)}$ is the matrix of population distribution in the year the epidemic starts (t_0_) over each

l, k, a stratum and it is defined in terms of: (i) $\varphi_{f(l)}$ and $\varphi_{m(l)}$which are the proportion of females and males respectively in each risk activity group; and (ii) f_a_ which is the proportion of the population in each year of age, with $\sum_{a=1}^{100} f_{a}=1$. The parameter f_cm_ gives the fraction of males who are circumcised.

The total number in the population (N) and $\Psi_{\left( l,k,p,a \right)}$ are given by:

$$N=\sum_{l=1}^{3} \sum_{k=1}^{3} \sum_{p=1}^{7} \sum_{a=1}^{100} \left( X_{l,k,a}^{0,p}+X_{l,k,a}^{1,p}+X_{l,k,a}^{2,p}+X_{l,k,a}^{3,p}+X_{l,k,a}^{4,p}+X_{l,k,a}^{5,p}+X_{l,k,a}^{6,p}+X_{l,k,a}^{7,p}+X_{l,k,a}^{8,p}+X_{l,k,a}^{9,p} \right)$$

(4)

$$\Psi_{\left( l,k,p,a \right)}=\left\{ \begin{aligned} \frac{1}{2}\varphi_{f(l)}f_{p} f_{a} any l; k=1;any p; any a; \\ \frac{1}{2}\varphi_{m(l)}f_{a}\left( 1-f_{cm} \right) any l; k=2; p=1; any a; \\ \frac{1}{2}\varphi_{m(l)}f_{a}f_{cm} any l; k=3;p=1; any a; \end{aligned} \right.$$

(5)

Individuals enter the population as susceptible at birth (i.e. age zero), the distribution of whom is defined by the population distribution matrix over each l, k and p stratum ($\Psi_{\left( l,k,p,1 \right)}$), given by:

$$\Psi_{\left( l,k,1,1 \right)}=\left\{ \begin{aligned} \frac{1}{2}\varphi_{f\left( l \right)} any l; k=1;p=1 (no method); a=1 (0 years); \\ \frac{1}{2}\varphi_{m\left( l \right)}\left( 1-f_{cm} \right) any l; k=2;p=1 (no method); a=1 (0 years); \\ \frac{1}{2}\varphi_{m\left( l \right)}f_{cm} any l; k=3; p=1 (no method); a=1 (0 years); \end{aligned} \right.$$

(6)

Age-specific fertility rates (b_a,t_) and age- and sex-specific non-AIDS mortality rates (μ_k,a,t_) are taken from the ASSA 2008 model and are updated each year from 1985 to 2025 as estimated by that model.[^11^](#_ENREF_11) AIDS-related mortality is modeled explicitly (Table S1). The South African population distribution by age in 1985 (f _a_) is taken from the same source. The fraction of the female population using each contraceptive method ($f_{p})$ is based on the South African National HIV Prevalence, Incidence and Behaviour Survey, 2012.[^12^](#_ENREF_12)

# 1.4 HIV transmission and sexual mixing

##### *Force of infection*

The force of infection is the per capita rate at which susceptible individuals acquire infection. Following previous work, the per capita force of infection $\lambda_{l,k,A}^{p}$ is the force of infection experienced by individuals of each contraceptive group, risk group, sex, circumcision status (if male) and five year age group from the infected population of the opposite sex at a given time. Characteristics of an individual (p, l, k and A (where A is five year age group)) are distinguished from those of their sexual partners by means of a prime (i.e. p’, l’, k’ and A’). The force of infection is calculated by five year age group and then applied to each single year of age in that group.

The force of infection depends on the pattern of partnership formation between different risk and five year age groups and on the probability of transmission per partnership, and is defined as:

For women:

$$\lambda_{l,1,A}^{p}=\sum_{l'} \sum_{k'} \sum_{p'} \sum_{A'} \sum_{s'} \left[ C_{g,A,l}\rho_{g,A,l,A^{'},l^{'}}\left( \frac{X_{l^{'},k^{'},A^{'}}^{s^{'}, p^{'}}}{\sum_{k^{'}=2}^{3} \sum_{p'} \sum_{s'} X_{l^{'},k^{'},A^{'}}^{s^{'},p^{'}}} \right)Z_{l,1,p,s^{'},l^{'},k^{'},p^{'}} \right]$$

For men:

$$\lambda_{l,k,A}^{p}=\sum_{l'} \sum_{p'} \sum_{A'} \sum_{s'} \left[ C_{g,A,l}\rho_{g,A,l,A^{'},l^{'}}\left( \frac{X_{l^{'},1,A'}^{s^{'}, p^{'}}}{\sum_{p'} \sum_{s'} X_{l^{'},1,A^{'}}^{s^{'},p^{'}}} \right)Z_{l,k,p,s^{'},l^{'},1,p^{'}} \right]$$

(7)

##### *Probability of transmission per partnership*

The probability of transmission per partnership depends on (i) the probability of transmission per sex act, and (ii) the number of sex acts during the partnership (which depends on the risk group of each partner). The probability of transmission per sex act depends on an individual’s circumcision status (if male), in addition to their partner’s state of HIV infection (including ART use), circumcision status (if male), DMPA use of the female partner and the degree of condom use in the partnership (which depends on the risk group of each partner).

A baseline transmission probability from uncircumcised males to females is assumed (β_0_). The difference in acquisition and transmission per sex act for other factors (e.g. stage of infection) is specified with respect to this baseline transmission probability using a multiplicative factor. The probability of HIV transmission per sex act is given by $\beta_{p,k}^{s^{'}k^{'}}$and depends on: s’ (partner’s HIV status), k’ (partner’s circumcision status (if male)), p (individual’s contraception status, i.e. DMPA use / nonuse) and k (individual’s circumcision status (if male)). The probability of transmission from males to females is assumed to be identical to that for transmission from females to males. Male circumcision is assumed to reduce the risk of acquisition but not onward transmission.

The number of sex acts in a partnership depends on the risk group of both partners and is given by the matrix n_sex_(l,l’). Condom use is modeled as a proportion of sex acts in which condoms are used via the matrix CU(l,l’), which defines condom use in a partnership between an individual’s risk group l and their partner’s risk group l’, modulated by any increase in condom use due to changes over time $\bar{q}$(t). The efficacy of condoms is given as ϖ.

The probability of transmission per partnership $Z_{l,k,p,s^{'},l^{'},k^{'},p^{'}}$ is defined as:

For women and for uncircumcised men:

$$Z_{l,k,1,s^{'},l^{'},1,p^{'}}=1-\left( \left( 1-\beta_{1,k}^{s^{'}k^{'}}{\varpi)}^{X} \right)\left( (1-\beta_{1,k}^{s^{'}k^{'}})^{\bar{X}} \right. \right)$$

For circumcised men:

$$Z_{l,3,1,s^{'},l^{'},k^{'},p^{'}}=1-\left( \left( 1-\beta_{1,3}^{s^{'}k^{'}}{\varpi)}^{X} \right)\left( (1-\beta_{1,3}^{s^{'}k^{'}})^{\bar{X}} \right. \right)$$

(8)

Where:

$$X=CU(l,l^{'})\bar{q}_{(t)}n_{sex}\left( l,l^{'} \right)$$

$$\bar{X}=\left( 1-CU(l,l^{'})\bar{q}_{\left( t \right)} \right)n_{sex}\left( l,l^{'} \right)$$

(9)

That is, X is the number of sex acts protected by condoms in a partnership between an individual of risk group l and their partner of risk group l’ and $\bar{X}$ is the number of sex acts not protected by condoms in a partnership between an individual of risk group l and their partner of risk group l’.

##### *Sexual mixing*

The mixing pattern is defined with respect to sex, five-year age group and behavioural risk group. The proportion of sexual partnerships that an individual of sex g (where g=1 refers to females and g=2 to males), 5 year age group A and risk group l forms with an individual of the opposite sex, age group A’ and risk group l’, is given by $\rho_{g,A,l,A^{'}l^{'}}$, and is defined as:

$$P_{1,A,l,A^{'}l^{'}}=\varepsilon_{A}\varepsilon_{l}\left( \delta_{A,A^{'}}\delta_{l,l^{'}} \right)+\left( 1-\varepsilon_{A} \right)\varepsilon_{l}\left( \delta_{l,l^{'}}\frac{C_{2,A^{'},l^{'}}\sum_{k^{'}=2}^{3} \sum_{p^{'}} \sum_{s^{'}} X_{l^{'},k^{'},A^{'}}^{s^{'},p^{'}}}{\sum_{A^{'}} C_{2,A^{'},l^{'}}\sum_{k^{'}=2}^{3} \sum_{p^{'}} \sum_{s^{'}} X_{l^{'},k^{'},A^{'}}^{s^{'},p^{'}}} \right)+\varepsilon_{A}\left( 1-\varepsilon_{l} \right)\left( \delta_{A,A^{'}}\frac{C_{2,A^{'},l^{'}}N_{g^{'}}\left( A^{'},l^{'} \right)}{\sum_{l^{'}} C_{2,A^{'},l^{'}}\sum_{k^{'}=2}^{3} \sum_{p^{'}} \sum_{s^{'}} X_{l^{'},k^{'},A^{'}}^{s^{'},p^{'}}} \right)+\left( 1-\varepsilon_{A} \right)\left( 1-\varepsilon_{l} \right)\left( \frac{C_{2,A^{'},l^{'}}N_{g^{'}}\left( A^{'},l^{'} \right)}{\sum_{A^{'}} \sum_{l^{'}} C_{2,A^{'},l^{'}}\sum_{k^{'}=2}^{3} \sum_{p^{'}} \sum_{s^{'}} X_{l^{'},k^{'},A^{'}}^{s^{'},p^{'}}} \right)$$

$$P_{2,A,l,A^{'}l^{'}}=\varepsilon_{A}\varepsilon_{l}\left( \delta_{A,A^{'}}\delta_{l,l^{'}} \right)+\left( 1-\varepsilon_{A} \right)\varepsilon_{l}\left( \delta_{l,l^{'}}\frac{C_{1,A^{'},l^{'}}\sum_{p^{'}} \sum_{s^{'}} X_{l^{'},1,A^{'}}^{s^{'},p^{'}}}{\sum_{A^{'}} C_{1,A^{'},l^{'}}\sum_{p^{'}} \sum_{s^{'}} X_{l^{'},1,A^{'}}^{s^{'},p^{'}}} \right)+\varepsilon_{A}\left( 1-\varepsilon_{l} \right)\left( \delta_{A,A^{'}}\frac{C_{1,A^{'},l^{'}}\sum_{p^{'}} \sum_{s^{'}} X_{l^{'},1,A^{'}}^{s^{'},p^{'}}}{\sum_{l^{'}} C_{1,A^{'},l^{'}}\sum_{p^{'}} \sum_{s^{'}} X_{l^{'},1,A^{'}}^{s^{'},p^{'}}} \right)+\left( 1-\varepsilon_{A} \right)\left( 1-\varepsilon_{l} \right)\left( \frac{C_{1,A^{'},l^{'}}\sum_{p^{'}} \sum_{s^{'}} X_{l^{'},1,A^{'}}^{s^{'},p^{'}}}{\sum_{A^{'}} \sum_{l^{'}} C_{1,A^{'},l^{'}}\sum_{p^{'}} \sum_{s^{'}} X_{l^{'},1,A^{'}}^{s^{'},p^{'}}} \right)$$

Note: $\sum_{A^{'}} \sum_{l^{'}} \rho_{g,A,l,A^{'},l^{'}}=1$

(10)

The parameter C_g,A,l_ gives the mean number of partners in a year per individual of sex g in age group A and risk group l. The degree of assortativity in mixing with respect to age and with respect to risk group are given by ε_A_ and ε_l_, respectively. The identity matrix with respect to risk is given by δ_l,l’_ whereby:

$$\delta_{l,l^{'}}=\left\{ \begin{aligned} 1, if l=l^{'} \\ 0, if l\neq l^{'} \end{aligned} \right.$$

(11)

A discrepancy matrix $D_{A_{2},l_{2},A_{1},l_{1}}$is defined to balance the number of sexual partnerships between males and females formed with respect to each age group and risk group, where A_2_ and l_2_ are the age and risk group of the male partner and A_1_ and l_1_ are the age and risk group of the female partner. It is calculated as follows:

$$D_{A_{2},l_{2},A_{1},l_{1}}= \frac{\rho_{2,A,l,A^{'}l^{'}}C_{2,A,l}\sum_{k=2}^{3} \sum_{p} \sum_{s} X_{l,k,A}^{s,p}}{\rho_{1,A,l,A^{'}l^{'}}C_{1,A,l}\sum_{p} \sum_{s} X_{l,1,A}^{s,p}}$$

(12)

The extent to which balancing of the number of sexual partnerships is male-driven is determined by parameter θ. When θ=0.5 the sexes compromise equally. Balancing the number of sexual partnerships is carried out with respect to both partners’ age and risk groups and is represented by:

$$\rho_{2,A,l,A^{'}l^{'}}\longrightarrow D_{A_{2},l_{2},A_{1},l_{1}}{}^{(\theta-1)}{\rho_{2,A,l,A^{'}l^{'}}}$$

$$\rho_{1,A,l,A^{'}l^{'}}\longrightarrow D_{A_{2},l_{2},A_{1},l_{1}}{}^{(\theta)}{\rho_{1,A,l,A^{'}l^{'}}}$$

(13)

**Table S2: Behavioural parameters and values**

| Parameter | Symbol | Value | Notes |
| --- | --- | --- | --- |
| Fraction of women in “low” risk group | ψf(1) | calibrated | Varies between model runs. |
| Fraction of women in “medium” risk group | ψf(2) | calibrated | Varies between model runs. Note all remaining women are assumed to be “high risk” |
| Fraction of men in low risk group | ψm(1) | calibrated | Varies between model runs. |
| Fraction of men in medium risk group | ψm(2) | calibrated | Varies between model runs. Note all remaining men are assumed to be “high risk” |

The number of sex acts per partnership depends on behavioural risk group. The “low” risk groups are intended to reflect long-term stable partnerships and these are assumed to have a high number of sex acts overall. A value of 100 sex acts each year is assumed based on reported frequency of sex in marital relationships in Southern Africa.[^13^](#_ENREF_13) Those in the higher risk groups tend to form more partnerships, but each of these partnerships comprises fewer sex acts and higher condom use. A value of two sex acts is assumed as a representative assumption of casual and commercial sex.

*β_0_ adjustment*

The model is run under different assumptions about the true HR for HIV risk among DMPA users. In the absence of an adjustment to the underlying HIV transmission probability, a higher HR would result in higher HIV prevalence, and therefore higher DALY burden. We required the baseline calibration to be independent of the true HR and therefore back-calculated β_0,_ the underlying transmission probability per sex act, for each model run, such that the same epidemic fit could be recreated using different values of the assumed true HR. Equation (14) shows the adjustment used to calculate β_0_.

$$\beta_{0}=\frac{\beta_{0 mean}}{(p.HR+1-p)}$$

(14)

**Table S3: Factors affecting transmission probability per sex act with respect to baseline transmission probability (β_0_)**

| Parameter | Symbol | Value | Source |
| --- | --- | --- | --- |
| Baseline transmission probability from an uncircumcised male in the asymptomatic stage of HIV infection to a female not using DMPA in a single act of unprotected sex | β_0_ | Variable | Calibrated for each model run. β_0_ is decreased for increasing HR such that the DALYs generated by each baseline model runs are approximately equal, regardless of the sampled HR.  The parameter is representative and captures impact of other risk factors not explicitly modelled such as infection with STIs other than HIV.[^14^](#_ENREF_14)^,^[^15^](#_ENREF_15) |
| Mean transmission probability per sex act across all women, representing a weighted mean of that among DMPA users and non-users. | β_0 mean_ | Calibrated | Calibrated under the assumption that HR=1 for DMPA users |
| Initial proportion of population using DMPA (aged 15-49) | p |  | Calculated within model |
| Factor increase in transmission: |  |  |  |
| To users of DMPA | $\beta_{3,1}^{s,k^{'}}$ | HR | Sampled from a distribution of hazard ratios. (See section 1.1) |
| From population with acute HIV infection | $\beta_{p,k}^{1,k^{'}}$ | 27 | [^7^](#_ENREF_7) |
| From population with chronic HIV infection and CD4 >350 cells/μL | $\beta_{p,k}^{2,k^{'}}$ | 1 | The baseline transmission probability is assumed to apply from the end of acute infection until the period of heightened infectiousness 19-10 months before death.[^7^](#_ENREF_7) |
| From population with chronic HIV infection and CD4 >200 cells/μL but <350 cells/μL | $\beta_{p,k}^{3,k^{'}}$ | 1.6 |  |
| From population in late infection | $\beta_{p,k}^{4,k^{'}}$ | 3.8 | [^7^](#_ENREF_7) |
| From population in pre-AIDS | $\beta_{p,k}^{5,k^{'}}$ | 3.8 |  |
| From population in AIDS | $\beta_{p,k}^{6,k^{'}}$ | 3.8 |  |
| From population on early ART | $\beta_{p,k}^{8,k^{'}}$ | 0.08 | [^8^](#_ENREF_8) |
| From population on late ART | $\beta_{p,k}^{10,k^{'}}$ | 0.08 | [^8^](#_ENREF_8) |
| From population who have dropped out of ART | $\beta_{p,k}^{11,k^{'}}$ | 3.56 | Estimated |
| From women | $\beta_{p,k}^{s^{'},1}$ | 1 | Transmission from males to females is assumed to be the same as that from females to males. |
| From uncircumcised men | $\beta_{p,k}^{s^{'},2}$ | 1 |  |
| From circumcised men | $\beta_{p,k}^{s^{'},3}$ | 1 | Assumes no effect of circumcision on  HIV transmission. |
| To circumcised men | $\beta_{p,3}^{s^{'},k^{1}}$ | 0.4 | Risk of HIV acquisition is 60% lower than among uncircumcised men[^16-18^](#_ENREF_16) |
| Condom efficacy |  | 0.1 | Assumes condoms provide 90% protection from HIV infection |

# 1.5 Male circumcision

The prevalence of circumcision changes over time to reflect the increase in male circumcision as has occurred in recent years according to a nationally representative survey and is projected to continue increasing in the future (Figure S2).[^12^](#_ENREF_12) The movement of uncircumcised men to circumcised classes is represent by the function $g\left( k \right)$, included in the equations below.

$$g\left( 1 \right)=0$$

$$g\left( 2 \right)= \frac{dX_{l,2,a}^{0,p}}{dt}-\eta_{C}$$

$$g\left( 3 \right)= \frac{dX_{l,3,a}^{0,p}}{dt}+\eta_{C}$$

(14)

The parameter $\eta_{C}$ gives the scale-up rate for male circumcision, which is a time-varying parameter based on the extent to which the current level of circumcision in the sexually active adult population matches the data on circumcision prevalence. Movement from uncircumcised to circumcised classes occurs at age 15 to represent circumcision that has occurred after birth but before entering the sexually active population. In the model, the rate of HIV acquisition for circumcised men is reduced by 60%.[^16-18^](#_ENREF_16)

# 1.6 Antiretroviral Treatment

ART can be initiated for the population with four programme types, specified with different initiation rules, as described above. A drop-out rate of 0.02 is assumed, regardless of the CD4 level at which ART is initiated. A rate of progressing to AIDS of 0.105 is assumed for those initiating ART below 200 cells/μL. The survival probability (p) of 0.9 reported by Mahy *et al.*[^19^](#_ENREF_19) was converted to a per capita mortality rate (r), using: p =1 - e^-rt^ . A rate of progressing to AIDS of 0.013 is assumed for those initiating ART above 200 cells/μL. A crude death rate of 1.3 deaths per 100 person years had been reported among individuals receiving early ART in the USA and Canada.[^20^](#_ENREF_20) The number of individuals receiving ART is calibrated to the total number of people on ART in South Africa (Figure S3).[^21^](#_ENREF_21)^,^[^22^](#_ENREF_22)

**1.7 Contraception**

We model the use of different contraceptives by splitting the female population into seven contraceptive compartments: no method, oral hormonal contraceptives, DMPA, norethisterone enanthate (NET-EN), copper intrauterine device (IUD), female sterilisation, implant and ‘other’ methods.

Condom use is modelled separately to female-controlled contraceptives; it is used to impact upon the rate of HIV transmission (section 1.4).

**Table S4. Contraceptive efficacy and continuation rates for methods used in the model.**

| Contraceptive | Efficacy (typical use) | One-year discontinuation rates (%) | Source |
| --- | --- | --- | --- |
| No method | 15% | Determined by uptake of other methods | [^23^](#_ENREF_23) |
| Combined oral contraceptive | 91% | 33 | [^23^](#_ENREF_23) |
| DMPA | 94% | 44 | [^23^](#_ENREF_23) |
| NET-EN | 94% | 44 | Assumed to be the same as DMPA |
| Copper IUD | 99.2% | 22 | [^23^](#_ENREF_23) |
| Female sterilisation | 99.5% | 0 | [^23^](#_ENREF_23) |
| Implant | 99.5% | 16 | [^23^](#_ENREF_23) |
| Other methods | 83.9% | 33 (assumed to be the same as oral contraceptives) | average of the efficacies of: male sterilisation, withdrawal, fertility awareness and male condoms[^23^](#_ENREF_23) |

The equations describing movement between contraceptive classes are given below.

$$q\left( p \right)= {{X_{l,k,a}^{s,p}}_{15-49}}_{(t+1)}- {{X_{l,k,a}^{s,p}}_{15-49}}_{(t)}$$

For p = 2:8

${{X_{l,k,a}^{s,p}}_{15-49}}_{(t+1)}= {{X_{l,k,a}^{s,p}}_{15-49}}_{(t)}+ \eta_{p}{{X_{l,k,a}^{s,1}}_{15-49}}_{(t)}- \sigma_{p}{{X_{l,k,a}^{s,p}}_{15-49}}_{(t)}$

For p = 1 (no method)

${{X_{l,k,a}^{s,1}}_{15-49}}_{(t+1)}= {{X_{l,k,a}^{s,1}}_{15-49}}_{(t)}+ \sum_{p=2}^{7} \sigma_{p}{{X_{l,k,a}^{s,p}}_{15-49}}_{(t)}- \sum_{p=2}^{7} \eta_{p}{{X_{l,k,a}^{s,1}}_{15-49}}_{(t)}$

(15)

Women move away from contraceptives to the “no method” compartment based on the discontinuation rates ($\sigma_{p}$, Table S4). The rate of uptake $\eta_{p}$ of each method is calibrated such that contraceptive prevalences approximate those reported in the South African National HIV Prevalence, Incidence and Behaviour Survey, 2012.[^12^](#_ENREF_12)

# 1.8 Reproductive health outcomes

*Maternal mortality*

Increases over time in the maternal mortality rate (MMR) whilst the HIV epidemic was growing (as seen in the Global Burden of Disease study[^24^](#_ENREF_24)) may mask declines in the MMR among HIV-negative women that may be expected given potential increases in access to and improved quality of health services. Estimates of MMR by HIV status are not available. The earliest MMR estimate in 1990 is therefore taken as an estimate of the MMR for HIV-negative women, as overall HIV prevalence was still relatively low in South Africa. For each simulation, the MMR for 1990 is sampled from a log-normal distribution based on a mean of 108 (95% uncertainty interval: 74 -159).[^25^](#_ENREF_25) MMR is assumed to remain constant between 1985 and 1990. To estimate a decline in the MMR among HIV-negative women in South Africa between 1990 and 2013 the rate of decline seen in the North Africa and Middle East region was applied to the MMR in South Africa. The rate of decline for North Africa and the Middle East was used because these are predominantly low and middle income countries (LMIC) but HIV prevalence in the region is below 1%, and therefore HIV has had a minimal impact on MMR decline.[^26^](#_ENREF_26) HIV-positive women experience MMR at a rate increased by a factor of 8.5 to reflect available evidence for increased risk of maternal mortality in this group. HIV-positive women receiving ART are assumed to experience the same MMR as HIV-negative women. This joint distribution for MMR is used for each set of comparable scenarios. The uncertainty intervals presented throughout the results represent 90% of model variation from both variation in the MMR and variation in sampled HIV-related parameters.

In addition to maternal mortality, there are a number of other complications of pregnancy included in our model. We include the risk of severe post-partum haemorrhage, maternal sepsis, eclampsia, obstructed labour and unsafe abortion. In each case, we only include the non-fatal injurious associated outcomes, as the associated mortality is included in the maternal mortality component of the model. These outcomes are described in Table 1 (main paper). Due to lack of available data there are also a number of complications of pregnancy that are not included in the model, including mastitis, perineal tears and urinary tract infections.

**Table S5. Parameters for reproductive health outcomes**

| Parameter | Value | Source |
| --- | --- | --- |
| MMR | Sampled from log-normal distribution. Mean 108 (72-159) | [^25^](#_ENREF_25) |
| Multiplicative factor applied to μ_mat(t)_ to reflect the increase in maternal mortality rate experienced by HIV-positive women not on ART | 8.5 (Calibrated within the range 3.98-10.29) | 6.40 (3.98-10.29)[^24^](#_ENREF_24) |
| Proportion of unintended pregnancies that end in abortion | 0.24 | Southern Africa estimate[^27^](#_ENREF_27) |
| Proportion of abortions that are unsafe | 0.2 | Reduced from estimate of 0.265 for Southern Africa,[^28^](#_ENREF_28) since South Africa has legal abortions. |
| Proportion of unsafe abortions resulting in reproductive tract infections that lead to secondary infertility | 0.12 | [^29^](#_ENREF_29) |

**1.9 Model calibration**

The per sex act probability of HIV transmission, sex-specific rates of mixing between different behavioural risk groups, the size of these risk groups for each sex and the start time of the epidemic were calibrated as these are difficult to reliably estimate empirically. This was done using data on age and sex-specific HIV prevalence, total HIV incidence and incidence in high risk women. We ran the model 20,000 times and used a filtration method to select 100 acceptable epidemic fits (Table S6). All model runs were then sampled from this set of 100.

The fitted sex-specific contact rates are multiplied by an age-specific cofactor, which was hand-fitted to improve the model calibration.

Rates of contraceptive uptake were calibrated to fit contraceptive data. To account for the decrease in average fertility due to the addition of contraceptive classes, the baseline fertility rate was increased and calibrated using population size data.

**Table S6. Calibrated parameters**

| **Parameter** | | **Median estimate** | **Low estimate** | | **High estimate** |
| --- | --- | --- | --- | --- | --- |
| HIV transmission probability per sex act | | 1.2 x 10^-3^ | 4.9 x 10^-4^ | | 2.2 x 10^-3^ |
| HIV epidemic start year | | 1984.1 | 1980.0 | | 1988.0 |
| Proportion in each risk group (women) | Low | 0.2129 | 0.0013 | | 0.8731 |
|  | High | 0.4054 | 0.0113 | | 0.7440 |
|  | FSW | 0.2749 | 0.0052 | | 0.9869 |
| Proportion in each risk group (men) | Low | 0.1642 | 0.0000 | | 0.8207 |
|  | Medium | 0.5007 | 0.0092 | | 0.8766 |
|  | High | 0.2873 | 0.0060 | | 0.9325 |
| Mean partner change rate (women) | Low | 1.6 | 0.0028 | | 7.2 |
|  | High | 4.3 | 0.12 | | 8.0 |
|  | FSW | 68 | 31 | | 120 |
| Mean partner change rate (men) | Low | 2.2 | 0.012 | | 7.7 |
|  | Medium | 3.6 | 0.048 | | 7.9 |
|  | High | 64 | 30 | | 120 |
| Multiplicative cofactor for 5-year age groups (hand fitted) |  | Women | | Men | |
|  | 15-19 | 2.40 | | 1.85 | |
|  | 20-24 | 1.95 | | 0.80 | |
|  | 25-29 | 1.50 | | 1.00 | |
|  | 30-34 | 1.10 | | 1.50 | |
|  | 35-39 | 1.35 | | 0.25 | |
|  | 40-44 | 1.40 | | 0.30 | |
|  | 45-49 | 1.50 | | 0.20 | |
|  | 50-54 | 1.50 | | 0.20 | |

1. **Supplementary Results**

**2.1 DALYs averted**

**Table S7. Total DALYs averted over 20 years (thousands).**Includes net HIV-related and reproductive health DALYs. Uncertainty intervals represent 90% of variability in model outputs.

|  | Magnitude of migration away from DMPA | Contraceptive replacement scenario | | | | | |
| --- | --- | --- | --- | --- | --- | --- | --- |
|  |  | 1. 100% switch to alternative with comparable efficacy | 2. 100% switch to implant | 3. 90% redistribute among other methods proportionately; 10% move to no method | 4. 80% redistribute among other methods proportionately; 20% move to no method | 5. 90% redistribute among other methods disproportionately in favour of NET-EN (OR=2); 10% move to no method | 6. 90% redistribute among other methods disproportionately in disfavour of NET-EN (OR=0.5); 10% move to no method |
| HR = 1.0 | Soft change | 0 (0 – 0) | 3.4 (3.2 – 3.6) | -12.5 (-11.8 – -13.4) | -23.3 (-21.6 – -25.1) | -12.4 (-11.8 – -13.2) | -12.6 (-12.0 – -13.5) |
|  | Medium change | 0 (0 – 0) | 8.1 (7.6 – 8.7) | -12.5 (-11.9 – -13.4) | -23.9 (-22.0 – -25.7) | -12.5 (-11.9 – -13.4) | -12.4 (-11.8 – -13.4) |
|  | Hard change | 0 (0 – 0) | 10.0 (9.3 – 10.7) | -13.7 (-13.0 – -14.9) | -28.8 (-27.3 – -31.0) | -13.6 (-12.9 – -14.8) | -13.9 (-13.1 – -15.1) |
| HR = 1.1 | Soft change | 28.4 (22.8 – 34.6) | 31.8 (26.1 – 37.9) | 16.9 (10.7 – 23.3) | 6.6 (-0.5 – 13.6) | 17.0 (10.9 – 23.3) | 16.8 (10.6 – 23.2) |
|  | Medium change | 72.1 (58.3 – 87.0) | 80.2 (66.7 – 94.9) | 59.6 (74.6 – 45.4) | 48.1 (33.2 – 63.8) | 59.6 (45.4 – 74.5) | 59.6 (45.3 – 74.8) |
|  | Hard change | 72.1 (58.3 – 87.0) | 82.0 (68.2 – 97.3) | 58.4 (44.5 – 72.9) | 43.3 (29.3 – 57.2) | 58.5 (44.5 – 73.0) | 58.2 (44.3 – 72.7) |
| HR = 1.2 | Soft change | 55.9 (44.9 – 68.1) | 59.3 (48.2 – 71.4) | 45.4 (33.6 – 58.0) | 35.5 (23.2 – 48.8) | 45.4 (33.7 – 57.9) | 45.4 (33.6 – 58.0) |
|  | Medium change | 141 (115 – 171) | 150 (123 – 179) | 129 (102 – 158) | 117 (89.8 – 148) | 129 (102 – 158) | 129 (102 - 159) |
|  | Hard change | 141 (115 – 171) | 151 (124 – 181) | 128 (101 – 157) | 113 (85.8 – 141) | 128 (101 – 157) | 128 (101 – 157) |
| HR = 1.3 | Soft change | 82.6 (66.4 – 101) | 85.9 (69.7 – 104) | 73.0 (55.9 – 916) | 63.5 (45.8 – 83.0) | 72.8 (55.8 – 91.4) | 73.0 (91.7 – 55.9) |
|  | Medium change | 208 (169 – 252) | 216 (177 – 260) | 196 (156 – 239) | 184 (144 – 228) | 196 (156 – 239) | 196 (156 – 239) |
|  | Hard change | 208 (169 – 252) | 218 (179 – 262) | 195 (155 – 238) | 180 (140 – 222) | 195 (155 – 238) | 195 (155 – 237) |

**2.4 Sensitivity analysis**

A sensitivity analysis was carried out on the time taken for transition to the new method mix. Increasing the transition time from three to six years showed the same pattern of results, with the six-year transition period giving absolute outcomes 60-80% lower over a five-year period and 5-20% lower over twenty years (data not shown).

**References**

1. Cremin I, Alsallaq R, Dybul M, Piot P, Garnett G, Hallett TB. The new role of antiretrovirals in combination HIV prevention: a mathematical modelling analysis. *AIDS* 2013; **27**(3): 447-58.

2. Beacroft L, Smith JA, Hallett TB. What impact could DMPA use have had in South Africa and how might its continued use affect the future of the HIV epidemic? *Journal of the International AIDS Society* 2019; **22**(11): e25414.

3. Smith JA, Anderson S-J, Harris KL, et al. Maximising HIV prevention by balancing the opportunities of today with the promises of tomorrow: a modelling study. *The Lancet HIV* 2016; **3**(7): e289-e96.

4. Anderson R, May R. Infectious Diseases of Humans: Dynamics and Control. Oxford: Oxford Science Publications; 1991.

5. Garnett GP, Anderson RM. Factors controlling the spread of HIV in heterosexual communities in developing countries: patterns of mixing between different age and sexual activity classes. *Philosophical Transactions of the Royal Society of London Series B: Biological Sciences* 1993; **342**(1300): 137-59.

6. Garnett GP, Anderson RM. Sexually Transmitted Diseases And Sexual Behavior: Insights From Mathematical Models. *The Journal of Infectious Diseases* 1996; **174**(Supplement_2): S150-S61.

7. Hollingsworth TD, Anderson Roy M, Fraser C. HIV-1 Transmission, by Stage of Infection. *The Journal of Infectious Diseases* 2008; **198**(5): 687-93.

8. Donnell D, Baeten JM, Kiarie J, et al. Heterosexual HIV-1 transmission after initiation of antiretroviral therapy: a prospective cohort analysis. *The Lancet* 2010; **375**(9731): 2092-8.

9. Cohen MS, Chen YQ, McCauley M, et al. Prevention of HIV-1 Infection with Early Antiretroviral Therapy. *New England Journal of Medicine* 2011; **365**(6): 493-505.

10. Lodi S, Phillips A, Touloumi G, et al. Time From Human Immunodeficiency Virus Seroconversion to Reaching CD4+ Cell Count Thresholds <200, <350, and <500 Cells/mm3: Assessment of Need Following Changes in Treatment Guidelines. *Clinical Infectious Diseases* 2011; **53**(8): 817-25.

11. Dorrington R, Johnson L, Budlender D. ASSA2008 AIDS and Demographic Models User Guise (beta version). Cape Town: Centre for Actuarial Research, University of Cape Town, 2010.

12. Shisana O, Rehle T, Simbayi LC, et al. South African National HIV Prevalence, Incidence and Behaviour Survey, 2012. Cape Town, 2014.

13. Brown MS. Coitus, the proximate determinant of conception: inter-country variance in sub-Saharan Africa. *J Biosoc Sci* 2000; **32**: 145-59.

14. Boily MC, Baggaley RF, Wang L, et al. Heterosexual risk of HIV-1 infection per sexual act: systematic review and meta-analysis of observational studies. *Lancet Infectious Diseases* 2009; **9**(2): 118-29.

15. Wawer MJ, Gray RH, Sewankambo NK, et al. Rates of HIV-1 Transmission per Coital Act, by Stage of HIV-1 Infection, in Rakai, Uganda. *The Journal of Infectious Diseases* 2005; **191**(9): 1403-9.

16. Bailey RC, Moses S, Parker CB, et al. Male circumcision for HIV prevention in young men in Kisumu, Kenya: a randomised controlled trial. *The Lancet* 2007; **369**(9562): 643-56.

17. Auvert B, Taljaard D, Lagarde E, Sobngwi-Tambekou J, Sitta R, Puren A. Randomized, Controlled Intervention Trial of Male Circumcision for Reduction of HIV Infection Risk: The ANRS 1265 Trial. *PLoS Med* 2005; **2**(11): e298.

18. Gray RH, Kigozi G, Serwadda D, et al. Male circumcision for HIV prevention in men in Rakai, Uganda: a randomised trial. *The Lancet* 2007; **369**(9562): 657-66.

19. Mahy M, Lewden C, Brinkhof MWG, et al. Derivation of parameters used in Spectrum for eligibility for antiretroviral therapy and survival on antiretroviral therapy. *Sexually Transmitted Infections* 2010; **86**(Suppl 2): ii28-ii34.

20. Kitahata MM, Gange SJ, Abraham AG, et al. Effect of Early versus Deferred Antiretroviral Therapy for HIV on Survival. *New England Journal of Medicine* 2009; **360**(18): 1815-26.

21. Johnson LF. Access to antiretroviral treatment in South Africa, 2004 - 2011. *2012* 2012; **13**(1): 6.

22. UNAIDS. AIDSInfo. 2018. aidsinfo.unaids.org (accessed 12th February 2019).

23. Trussell J. Contraceptive efficacy. In: Hatcher R, Trussell J, Nelson A, Cates Jr. W, Kowal D, Policar M, eds. Contraceptive Technology: Twentieth Revised Edition. New York: Ardent Media; 2011.

24. Kassebaum NJ, Bertozzi-Villa A, Coggeshall MS, et al. Global, regional, and national levels and causes of maternal mortality during 1990-2013: a systematic analysis for the Global Burden of Disease Study 2013. *The Lancet* 2014.

25. Trends in maternal mortality: 1990 to 2015: estimates by WHO, UNICEF, UNFPA, World Bank Group and the United Nations Population Division. Geneva, Switzerland: World Health Organization,, 2015.

26. Kassebaum NJ, Barber RM, Bhutta ZA, et al. Global, regional, and national levels of maternal mortality, 1990-2015: a systematic analysis for the Global Burden of Disease Study 2015. *The Lancet* 2016; **388**(10053): 1775-812.

27. Sedgh G, Bearak J, Singh S, et al. Abortion incidence between 1990 and 2014: global, regional, and subregional levels and trends. *The Lancet* 2016; **388**(10041): 258-67.

28. Ganatra B, Gerdts C, Rossier C, et al. Global, regional, and subregional classification of abortions by safety, 2010-14: estimates from a Bayesian hierarchical model. *The Lancet* 2017; **390**(10110): 2372-81.

29. Ahman E, Dolea C, Shah I. The global burden of unsafe abortion in the year 2000. Geneva: World Health Organisation, 2003.
